# Frequentist and Bayesian Predictive Inference for the Log-Logistic Distribution Under Progressive Type-II Censoring

**DOI:** 10.3390/e28040466

**Published:** 2026-04-18

**Authors:** Ziteng Zhang, Wenhao Gui

**Affiliations:** School of Mathematics and Statistics, Beijing Jiaotong University, Beijing 100044, China; 23271024@bjtu.edu.cn

**Keywords:** Log-Logistic distribution, progressive censoring data, bayes estimation, bayes prediction, MCMC sampling, simulation

## Abstract

This paper investigates the prediction of unobserved future failure times for the heavy-tailed Log-Logistic distribution under Progressive Type-II censoring. We first develop point and interval estimates for the unknown parameters using both frequentist maximum likelihood and Bayesian approaches. For predicting future failures, we derive three distinct point predictors: the Best Unbiased Predictor (BUP), the Conditional Median Predictor (CMP), and the Bayesian Predictor (BP). Corresponding prediction intervals are constructed using frequentist pivotal quantities, Bayesian Equal-Tailed Intervals (ETIs), and Highest Posterior Density (HPD) methods. The Bayesian procedures are implemented via Markov chain Monte Carlo (MCMC) sampling. We evaluate the finite-sample performance of the proposed methodologies through a Monte Carlo simulation study and further validate them using two real-world datasets, namely bladder cancer remission times and guinea pig survival times. The numerical results indicate that the proposed BP, particularly under the empirical prior, provides the most accurate and stable overall performance for point prediction, while the frequentist predictors become less reliable in extreme heavy-tailed settings. For interval prediction, the Bayesian HPD method consistently outperforms the alternatives, substantially reducing interval lengths for right-skewed data while maintaining the nominal coverage probability.

## 1. Introduction

In many engineering and biomedical systems, failure rates do not strictly follow a simple monotonic trend; instead, they frequently exhibit a non-monotonic, unimodal trajectory. During the early operational phase, the hazard rate rises due to initial stress factors, reaches a finite peak, and eventually declines as the system stabilizes. This upside-down bathtub (UBT) pattern is widely observed in systems such as medical devices, rotating machinery, and communication components.

To evaluate system reliability and capture these UBT characteristics, researchers conventionally rely on life-testing experiments. However, the prohibitive cost and time constraints of such experiments often result in incomplete or censored failure data. Under these constraints, extracting predictive inference from limited observations becomes essential for assessing the remaining life of surviving units. Predicting future failure times provides practical guidance for engineering decisions, such as establishing warranty periods, optimizing spare parts inventory, and scheduling preventive maintenance.

From a statistical perspective, this translates into a formal prediction problem. Prediction problems are generally categorized into two types: one-sample prediction (OSP), where the future variables to be predicted originate from the same experiment and are correlated with the observed sample; and two-sample prediction (TSP), where the future variables come from an independent future sample. This paper focuses on the OSP framework, which aligns with the aforementioned life-testing scenarios, aiming to perform predictive inference for unobserved failure times using available censored data.

OSP has received considerable attention, with extensive theoretical frameworks developed under classical lifetime models. For instance, Lawless [1] provided a comprehensive treatment of prediction intervals for the Weibull and Exponential distributions, while Kundu and Raqab [2] addressed Bayesian Prediction for Type-II censored Weibull data. However, classical lifetime models may be restrictive when failure mechanisms exhibit non-monotonic hazard behavior or pronounced tail heterogeneity. The Weibull distribution is limited to monotone hazard rates and therefore cannot represent UBT-shaped hazards [3]. Although the Log-normal distribution is more flexible in this respect, models with more direct survival and quantile representations may be advantageous in censored settings from the standpoint of analytical tractability. This motivates consideration of alternative lifetime models, such as the Log-Logistic distribution, which can accommodate UBT-shaped hazard behavior and heavy-tailed survival patterns.

The Log-Logistic distribution serves as an effective alternative for modeling such non-monotonic hazard processes. Originally introduced by Fisk [4], this distribution is characterized by a shape parameter that allows it to generate either a strictly decreasing or a unimodal hazard rate, making it well-suited for products experiencing early wear-in failures followed by a recovery phase. Let the random variable *T* follow a Log-Logistic distribution with scale parameter α>0 and shape parameter β>0; its cumulative distribution function is

(1) $$F\left(t\right)=\frac{1}{1+{\left(\frac{t}{\alpha }\right)}^{-\beta }},\;t>0.$$

From this expression, the corresponding probability density function is

(2) $$f\left(t\right)=\frac{\beta }{\alpha }{\left(\frac{t}{\alpha }\right)}^{\beta -1}{\left[1+{\left(\frac{t}{\alpha }\right)}^{\beta }\right]}^{-2},\;t>0.$$

Furthermore, the survival (or reliability) function, which represents the probability that a unit survives beyond a specific time *t* (i.e., S(t)=P(T>t)), is essential for handling censored observations and has a highly tractable closed form:

(3) $$S\left(t\right)=1-F\left(t\right)=\frac{1}{1+{\left(\frac{t}{\alpha }\right)}^{\beta }},\;t>0,$$

and consequently, the hazard function, which quantifies the instantaneous failure rate at time *t* conditional on survival up to that time, takes the form

(4) $$h\left(t\right)=\frac{f(t)}{S(t)}=\frac{\frac{\beta }{\alpha }{\left(t/\alpha \right)}^{\beta -1}}{1+{\left(t/\alpha \right)}^{\beta }},\;t>0.$$

Its shape parameter allows for either strictly decreasing or unimodal hazard rates, while its heavy-tailed property accommodates long-lived components. These features make the Log-Logistic distribution a useful candidate for modeling lifetime data with non-monotonic failure behavior. In addition, its survival function S(t) and hazard function h(t) admit simple closed-form expressions, which facilitate likelihood-based inference and prediction under censoring.

Due to these properties, the Log-Logistic distribution is widely used across various fields. In reliability engineering, Nelson [5] analyzed general failure data, and Clavijo-Blanco et al. [6] applied it to distribution network reliability indices. Applications extend to seismic risk estimation [7], hydrology for flood frequency analysis [8], and economics for income inequality [4]. In the medical domain, it has been used as parametric regression baselines for time-to-event outcomes, including applications to cancer survival data [9,10].

Statistical inference for the Log-Logistic distribution was initially developed in the complete-sample setting. Fisk [4] established early foundational properties of the distribution. O’Quigley and Struthers [11] subsequently introduced logistic and Log-Logistic survival models and provided practical fitting tools for censored survival data. Classical estimation for the Log-Logistic model was further studied by Balakrishnan and Malik [12], who derived best linear unbiased estimators (BLUEs) for the location and scale parameters. From a Bayesian perspective, Al-Shomrani et al. [13] employed MCMC techniques for complete data, while Abbas and Tang [14] developed an objective Bayesian analysis based on reference and Jeffreys priors.

In reliability testing, complete failure data are rarely available, necessitating censored inference. The most fundamental frameworks are Type-I (time) and Type-II (failure) censoring. Inference for the Log-Logistic distribution under these classical schemes is well established: Howlader and Weiss [15] estimated survival functions under Type-II censoring, while Kantam et al. [16] developed reliability test plans under Type-I censoring. To balance the temporal and failure-count constraints of individual schemes, hybrid censoring integrates Type-I and Type-II mechanisms. For the Log-Logistic model, Hyun et al. [17] derived maximum likelihood estimators and asymptotic confidence intervals under hybrid censored data.

However, traditional and hybrid schemes generally restrict the withdrawal of surviving units to the experiment’s termination. To accelerate testing and optimize resource allocation, Progressive Type-II Censoring allows the withdrawal of surviving units at various stages during the experiment rather than only at the end. Consider an experiment with *n* independent units, and let $X_{i:m:n}$ denote the *i*-th observed failure time in a progressively censored sample of size *m*. Prior to the experiment, a progressive censoring scheme $R=(r_{1},r_{2},\ldots ,r_{m})$ is pre-specified, satisfying the sample size constraint $n=m+\sum_{i=1}^{m}r_{i}$. Under this scheme, when the first failure occurs at $X_{1:m:n}$, $r_{1}$ surviving units are randomly selected and removed. At the second failure $X_{2:m:n}$, $r_{2}$ surviving units are randomly removed, and the process continues iteratively until the *m*-th failure, $X_{m:m:n}$, where the experiment terminates and all remaining $r_{m}=n-m-\sum_{i=1}^{m-1}r_{i}$ survivors are removed.

This framework is highly advantageous in industrial settings, as the early withdrawn units can be repurposed or subjected to different screening tests, thereby reducing the total duration and cost. This scheme reduces to classical Type-II censoring when $r_{1}=\cdots =r_{m-1}=0$ and $r_{m}=n-m$; see Balakrishnan and Aggarwala [18] for a systematic treatment.

Extensive inference methodologies have been developed under Progressive Type-II Censoring for various models [19]. These include maximum-likelihood estimation and exact interval inference for the Weibull distribution [20]. For heavy-tailed models, interval estimation [21] and prediction intervals [22] were developed for the Burr-XII distribution, while predictive inference for the Pareto distribution under progressive Type-II censoring has also been investigated [23]. Research also covers models with non-monotonic hazards, such as the Chen [24], Generalized Exponential [25], and Inverse Weibull [26] distributions.

Despite these methodological developments, predictive inference for the Log-Logistic distribution under Progressive Type-II Censoring remains unexplored. Specifically, there is a lack of one-sample prediction methodologies that integrate both frequentist and Bayesian frameworks for this distribution. Addressing this is practically relevant, as combining the heavy-tailed Log-Logistic model with progressive censoring provides a flexible approach for analyzing long-duration reliability data. Consequently, this paper investigates parameter estimation and predictive inference for future failure times under the Log-Logistic distribution with Progressive Type-II Censoring.

The remainder of this paper is organized as follows. Section 2 derives the Maximum Likelihood Estimators (MLEs) and Asymptotic Confidence Intervals (ACIs) for the Log-Logistic parameters under Progressive Type-II censoring. Section 3 develops the Bayesian inference procedure, utilizing independent Gamma priors and a Metropolis–Hastings within Gibbs algorithm to obtain the Bayes estimates and Credible Intervals (CIs). Section 4 derives three point predictors: the Best Unbiased Predictor (BUP), the Conditional Median Predictor (CMP), and the Bayesian Predictor (BP). Section 5 constructs the interval predictors, including frequentist pivotal prediction Intervals (PPIs), the Bayesian Equal-Tailed Intervals (ETIs), and the Highest Posterior Density (HPD) intervals. Section 6 evaluates the finite-sample performance of the proposed methods through a Monte Carlo simulation study. Section 7 applies the methodologies to two real-world datasets. Finally, Section 8 provides concluding remarks.

## 2. Frequentist Statistical Analysis

This section investigates the frequentist maximum likelihood estimation of the Log-Logistic distribution parameters α and β under progressive Type-II censoring. Let $x=\left(x_{1:m:n},x_{2:m:n},\ldots ,x_{m:m:n}\right)$, where $x_{1:m:n}\leq x_{2:m:n}\leq \cdots \leq x_{m:m:n}$, denote a progressively Type-II censored sample of size *m* obtained from a population with an initial sample size *n* under the censoring scheme $R=(r_{1},r_{2},\ldots ,r_{m})$. For notational simplicity, we write $x_{i:m:n}$ as $x_{i}$. Recall that the cumulative distribution function F(·) and probability density function f(·) of the underlying Log-Logistic distribution are given by Equations (1) and (2), respectively.

Conditioning on the censoring scheme R, it follows from Balakrishnan and Aggarwala [18] that the likelihood function of the progressively Type-II censored sample can be expressed as

(5) $$L\left(\alpha ,\beta |x\right)=C\prod_{i=1}^{m}\left[f\left(x_{i};\alpha ,\beta \right){\left(1-F(x_{i};\alpha ,\beta )\right)}^{r_{i}}\right],$$

where $C=\prod_{j=1}^{m}(n-\sum_{i=1}^{j-1}(r_{i}+1))$ is a constant independent of the unknown parameters.

Substituting the specific forms of the Log-Logistic distribution into Equation (5), the likelihood function can be explicitly written as

(6) $$L\left(\alpha ,\beta |x\right)=C\prod_{i=1}^{m}\left[\frac{\beta }{\alpha }{\left(\frac{x_{i}}{\alpha }\right)}^{\beta -1}{\left[1+{\left(\frac{x_{i}}{\alpha }\right)}^{\beta }\right]}^{-2}{\left(\frac{{\left(x_{i}/\alpha \right)}^{-\beta }}{1+{\left(x_{i}/\alpha \right)}^{-\beta }}\right)}^{r_{i}}\right].$$

Taking the natural logarithm of Equation (6) yields the corresponding log-likelihood function, up to an additive constant:

(7) $$\begin{array}{cc} 𝓁(\alpha ,\beta ) & \propto \sum_{i=1}^{m}[\ln\;\left(\frac{\beta }{\alpha }\right)+\left(\beta -1\right)\ln\;\left(\frac{x_{i}}{\alpha }\right)-2\ln\;\left(1+{\left(\frac{x_{i}}{\alpha }\right)}^{\beta }\right)\\ \; & \;\;+r_{i}\left(-\beta \ln\;\left(\frac{x_{i}}{\alpha }\right)-\ln\;\left(1+{\left(\frac{x_{i}}{\alpha }\right)}^{-\beta }\right)\right)]. \end{array}$$

Taking the partial derivatives of the log-likelihood with respect to α and β, and equating them to zero, we obtain the following score equations:

(8) $$\frac{\partial 𝓁(\alpha ,\beta )}{\partial \alpha }=\frac{\beta }{\alpha }\left(\sum_{i=1}^{m}\left(2+r_{i}\right)\frac{{\left(x_{i}/\alpha \right)}^{\beta }}{1+{\left(x_{i}/\alpha \right)}^{\beta }}-m\right)=0,$$

(9) $$\frac{\partial 𝓁(\alpha ,\beta )}{\partial \beta }=\frac{m}{\beta }+\sum_{i=1}^{m}\left(\ln x_{i}-\ln\alpha \right)-\sum_{i=1}^{m}\left(2+r_{i}\right)\frac{{\left(\frac{x_{i}}{\alpha }\right)}^{\beta }\left(\ln x_{i}-\ln\alpha \right)}{1+{\left(\frac{x_{i}}{\alpha }\right)}^{\beta }}=0.$$

The existence of the maximum likelihood estimators can be established by analyzing the behavior of the log-likelihood function at the boundaries of the parameter space $\Theta ={\left(0,\infty \right)}^{2}$. Specifically, 𝓁(α,β) is continuous on Θ and diverges to −∞ as either α or β approaches $0^{+}$ or *∞*. This asymptotic property implies that all upper-level sets of the log-likelihood function are closed and bounded. As demonstrated by Mäkeläinen et al. [27], such boundary behavior ensures that 𝓁(α,β) attains its global maximum within the interior of Θ, thereby confirming the existence of the MLEs.

Consequently, since the global maximum lies in the interior of the open set Θ and the log-likelihood function is differentiable, the MLEs must satisfy the score Equations (8) and (9). However, because these nonlinear equations lack closed-form analytical solutions, numerical procedures are required. Here, we employ the Newton–Raphson method to obtain the parameter estimates. Letting $\phi ={\left(\alpha ,\beta \right)}^{⊤}$, the update at the (k+1)-th iteration is given by

(10) $$\phi^{\left(k+1\right)}=\phi^{\left(k\right)}-H^{-1}\left(\phi^{\left(k\right)}\right)\nabla 𝓁\left(\phi^{\left(k\right)}\right),$$

where $\nabla 𝓁={\left(\frac{\partial 𝓁}{\partial \alpha },\frac{\partial 𝓁}{\partial \beta }\right)}^{⊤}$ is the gradient vector, and the Hessian matrix H(ϕ) is defined as

(11) $$H\left(\phi \right)=\left[\begin{array}{cc} \frac{\partial^{2}𝓁\left(\alpha ,\beta \right)}{\partial \alpha^{2}} & \frac{\partial^{2}𝓁\left(\alpha ,\beta \right)}{\partial \alpha \;\partial \beta }\\ \frac{\partial^{2}𝓁\left(\alpha ,\beta \right)}{\partial \beta \;\partial \alpha } & \frac{\partial^{2}𝓁\left(\alpha ,\beta \right)}{\partial \beta^{2}} \end{array}\right].$$

The specific components of the Hessian matrix are explicitly derived as follows:

(12) $$\begin{array}{cc} \frac{\partial^{2}𝓁\left(\alpha ,\beta \right)}{\partial \alpha^{2}} & =\frac{\beta }{\alpha^{2}}\left[m-\sum_{i=1}^{m}\left(2+r_{i}\right)\frac{{\left(\frac{x_{i}}{\alpha }\right)}^{\beta }}{1+{\left(\frac{x_{i}}{\alpha }\right)}^{\beta }}-\beta \sum_{i=1}^{m}\left(2+r_{i}\right)\frac{{\left(\frac{x_{i}}{\alpha }\right)}^{\beta }}{{\left(1+{\left(\frac{x_{i}}{\alpha }\right)}^{\beta }\right)}^{2}}\right], \end{array}$$

(13) $$\begin{array}{cc} \frac{\partial^{2}𝓁\left(\alpha ,\beta \right)}{\partial \beta^{2}} & =-\frac{m}{\beta^{2}}-\sum_{i=1}^{m}\left(2+r_{i}\right)\frac{{\left(\frac{x_{i}}{\alpha }\right)}^{\beta }{\left(\ln x_{i}-\ln\alpha \right)}^{2}}{{\left(1+{\left(\frac{x_{i}}{\alpha }\right)}^{\beta }\right)}^{2}}, \end{array}$$

(14) $$\begin{array}{cc} \frac{\partial^{2}𝓁\left(\alpha ,\beta \right)}{\partial \alpha \;\partial \beta } & =\frac{1}{\alpha }\left[-m+\sum_{i=1}^{m}\left(2+r_{i}\right)\frac{{\left(\frac{x_{i}}{\alpha }\right)}^{\beta }}{1+{\left(\frac{x_{i}}{\alpha }\right)}^{\beta }}+\beta \sum_{i=1}^{m}\left(2+r_{i}\right)\frac{{\left(\frac{x_{i}}{\alpha }\right)}^{\beta }\left(\ln x_{i}-\ln\alpha \right)}{{\left(1+{\left(\frac{x_{i}}{\alpha }\right)}^{\beta }\right)}^{2}}\right]. \end{array}$$

While the classical Newton–Raphson update operates on ϕ directly, it is highly sensitive to initial values and may fail to converge when the log-likelihood surface is flat. Furthermore, standard updates do not inherently prevent the parameters from crossing into invalid negative territories.

To enforce strict positivity constraints (α,β>0) and improve overall numerical stability, our computational implementation is executed in the unconstrained log-space $\psi ={\left(\ln\alpha ,\ln\beta \right)}^{⊤}$. The gradient and Hessian with respect to ψ can be readily evaluated via the chain rule from their original-space counterparts derived above. This modified Newton–Raphson procedure, which additionally incorporates OLS initialization, step-halving, and ridge regularization, is summarized in Algorithm 1.
**Algorithm 1** Modified Newton–Raphson procedure for Maximum Likelihood Estimation  1:**Input:** Progressively censored data $x=(x_{1},\ldots ,x_{m})$, censoring scheme $R=(r_{1},\ldots ,r_{m})$, total sample size *n*, gradient tolerance $\epsilon_{g}=10^{-6}$, step tolerance $\epsilon_{s}=10^{-8}$, maximum iterations K=200.  2:**Initialization:**  3:   Compute the empirical survival estimates $\hat{S}\left(x_{i}\right)$ via the Herd–Johnson product-limit method designed for progressive censoring:$$\hat{S}\left(x_{i}\right)=\prod_{j=1}^{i}\frac{n-\sum_{k=1}^{j-1}r_{k}-j+1}{n-\sum_{k=1}^{j-1}r_{k}-j+2},\;i=1,\ldots ,m.$$  4:   Construct the linearized regression variables based on the Log-Logistic odds ratio:$$V_{i}=\ln\left[\frac{1-\hat{S}\left(x_{i}\right)}{\hat{S}\left(x_{i}\right)}\right]\;\mathrm{and}\;U_{i}=\ln x_{i}.$$  5:   Perform ordinary least squares (OLS) regression of V against U to yield intercept $c_{0}$ and slope $c_{1}$.  6:   Extract initial values: $\beta^{\left(0\right)}=c_{1}$ and $\alpha^{\left(0\right)}=\exp\left(-c_{0}/c_{1}\right)$.  7:**Space Transformation:** To enforce positivity constraints strictly, map parameters to the unconstrained log-space:  8:   $\psi^{\left(0\right)}={\left(\ln\alpha^{\left(0\right)},\ln\beta^{\left(0\right)}\right)}^{⊤}$. Set iteration counter k=0.  9:**Repeat** until convergence or k=K:10:   Evaluate the gradient vector $g_{\psi }^{\left(k\right)}=\nabla 𝓁\left(\psi^{\left(k\right)}\right)$ and the Hessian matrix $H_{\psi }^{\left(k\right)}=\nabla^{2}𝓁\left(\psi^{\left(k\right)}\right)$.11:   **Regularization:** If $\left|\det(H_{\psi }^{\left(k\right)})\right|$ is near zero, apply ridge regularization: $H_{\psi }^{\left(k\right)}\leftarrow H_{\psi }^{\left(k\right)}+\lambda I$, where $\lambda =10^{-6}$.12:   Compute the full Newton step: $\Delta \psi ={\left[H_{\psi }^{\left(k\right)}\right]}^{-1}g_{\psi }^{\left(k\right)}$.13:   **Backtracking Line Search (Step-halving):**14:      Set $\psi_{test}^{\left(k+1\right)}=\psi^{\left(k\right)}-\Delta \psi$.15:      **While** $𝓁\left(\psi_{test}^{\left(k+1\right)}\right)<𝓁\left(\psi^{\left(k\right)}\right)$ and halving steps <25 **do**:16:         Δψ←Δψ/217:         $\psi_{test}^{\left(k+1\right)}=\psi^{\left(k\right)}-\Delta \psi$18:      **End While**19:   **Update:** $\psi^{\left(k+1\right)}=\psi_{test}^{\left(k+1\right)}$.20:   **Check Convergence:** If $\left|\right|g_{\psi }^{\left(k\right)}{\left|\right|}_{2}<\epsilon_{g}$ and ${\left||\Delta \psi |\right|}_{2}<\epsilon_{s}$, terminate loop.21:   k←k+122:**Output:** Transform back to the original parameter space: ${\hat{\alpha }}_{\mathrm{MLE}}=\exp\;\left(\psi_{1}^{\left(k\right)}\right)$ and ${\hat{\beta }}_{\mathrm{MLE}}=\exp\;\left(\psi_{2}^{\left(k\right)}\right).$

Under standard regularity conditions for censored lifetime models, and under an increasing-sample framework in which the total sample size n→∞, the number of observed failures $m=m_{n}\to \infty$, and the progressive censoring scheme remains asymptotically stable, the MLE $\hat{\phi }={\left({\hat{\alpha }}_{MLE},{\hat{\beta }}_{MLE}\right)}^{⊤}$ is asymptotically normal; see, e.g., Lawless [28] for general likelihood theory for censored lifetime data and Lin and Balakrishnan [29] for progressive Type-II censoring. Specifically,

(15) $$\sqrt{n}\left(\hat{\phi }-\phi \right)\overset{D}{\to }N_{2}\;\left(0,\;I_{0}^{-1}\left(\phi \right)\right),$$

where $I_{0}\left(\phi \right)$ denotes the limiting Fisher information matrix per unit.

In practical applications, since $I_{0}\left(\phi \right)$ depends on the unknown true parameter, the asymptotic variance–covariance matrix of $\hat{\phi }$ is commonly estimated by the inverse observed information matrix evaluated at the MLE, namely,

(16) $$\hat{\mathrm{Var}}\left(\hat{\phi }\right)\approx J_{n}^{-1}\left(\hat{\phi }\right),$$

where $J_{n}\left(\hat{\phi }\right)=-H\left(\hat{\phi }\right)$ is the full-sample observed information matrix.

Finally, let ${\hat{V}}_{11}$ and ${\hat{V}}_{22}$ denote the (1,1) and (2,2) diagonal elements of $J_{n}^{-1}\left(\hat{\phi }\right)$. Then the approximate 100(1−γ)% Wald confidence intervals for α and β are given by

(17) $${\hat{\alpha }}_{MLE}\pm z_{1-\gamma /2}\sqrt{{\hat{V}}_{11}},\;{\hat{\beta }}_{MLE}\pm z_{1-\gamma /2}\sqrt{{\hat{V}}_{22}},$$

where $z_{1-\gamma /2}$ is the (1−γ/2)-th quantile of the standard normal distribution.

## 3. Bayesian Statistical Analysis

This section derives the posterior distribution of the Log-Logistic distribution parameters based on a progressively Type-II-censored sample. While the parameters were treated as a vector $\phi ={\left(\alpha ,\beta \right)}^{⊤}$ in the previous section for asymptotic matrix derivations, we treat them component-wise in the Bayesian framework. This scalar representation is more natural for constructing independent prior distributions and formulating the sequential updating steps in the Markov Chain Monte Carlo scheme.

In Bayesian inference, the choice of prior distributions plays a key role in determining the flexibility and interpretability of the model. Since both α and β are positive-valued parameters, a natural and widely adopted specification is to assign independent Gamma priors to them, namely, α∼Gamma(a,b) and β∼Gamma(c,d), where the hyperparameters a,b,c,d>0 encode prior knowledge. Gamma priors enjoy several appealing properties, including support compatibility and computational convenience, making them prevalent in reliability literature. Moreover, in the absence of substantial prior information, weakly informative priors can be constructed by choosing relatively small hyperparameter values, thereby mitigating subjective influence.

The corresponding prior density functions are

(18) $$\pi_{\alpha }\left(\alpha |a,b\right)=\frac{b^{a}}{\Gamma (a)}\alpha^{a-1}e^{-b\alpha },\;\pi_{\beta }\left(\beta |c,d\right)=\frac{d^{c}}{\Gamma (c)}\beta^{c-1}e^{-d\beta }.$$

Assuming prior independence, the joint prior density is $\pi \left(\alpha ,\beta \right)=\pi_{\alpha }\left(\alpha |a,b\right)\times \pi_{\beta }\left(\beta |c,d\right)$. Hence, the normalized joint posterior distribution is given by

(19) $$\pi_{jp}\left(\alpha ,\beta |x\right)=\frac{L(\alpha ,\beta |x)\;\pi (\alpha ,\beta )}{\int_{0}^{\infty }\int_{0}^{\infty }L\left(\alpha ,\beta |x\right)\;\pi \left(\alpha ,\beta \right)\;d\alpha \;d\beta }.$$

Under the squared error loss (SEL) function, the Bayes estimator of any parametric function θ=g(α,β) is defined as its posterior mean:

(20) $$\theta_{\mathrm{BE}}=E_{\pi_{jp}}\left(\theta |x\right)=\frac{\int_{0}^{\infty }\int_{0}^{\infty }g\left(\alpha ,\beta \right)\;L\left(\alpha ,\beta |x\right)\;\pi \left(\alpha ,\beta \right)\;d\alpha \;d\beta }{\int_{0}^{\infty }\int_{0}^{\infty }L\left(\alpha ,\beta |x\right)\;\pi \left(\alpha ,\beta \right)\;d\alpha \;d\beta }.$$

Because the joint posterior distribution $\pi_{jp}\left(\alpha ,\beta |x\right)$ does not admit a closed-form normalization integral, explicit Bayes estimators are not available. We therefore resort to MCMC-based numerical methods. Given β and x, the full conditional kernel of α is

(21) $$\pi_{1}\left(\alpha |\beta ,x\right)\propto \alpha^{a-1-m\beta }\;e^{-b\alpha }\prod_{i=1}^{m}{\left[1+{\left(\frac{x_{i}}{\alpha }\right)}^{\beta }\right]}^{-(2+r_{i})},\;\alpha >0,$$

and, similarly, given α and x, the full conditional kernel of β is

(22) $$\pi_{2}\left(\beta |\alpha ,x\right)\propto \beta^{c+m-1}\;e^{-d\beta }\prod_{i=1}^{m}{\left(\frac{x_{i}}{\alpha }\right)}^{\beta }{\left[1+{\left(\frac{x_{i}}{\alpha }\right)}^{\beta }\right]}^{-(2+r_{i})},\;\beta >0.$$

Clearly, neither conditional distribution can be reduced to a familiar conjugate form. To address this, we adopt a Metropolis–Hastings-within-Gibbs MCMC scheme. To ensure unconstrained random-walk proposals, we reparameterize the variables to the real line via η=logα and ζ=logβ. Accounting for the Jacobian of the transformation ($e^{\eta }$ and $e^{\zeta }$), the full conditional distributions in the log-space are given by

(23) $$\begin{array}{cc} \pi_{\eta }\left(\eta |\zeta ,x\right) & \propto \pi_{1}\left(e^{\eta }|e^{\zeta },x\right)\cdot e^{\eta }, \end{array}$$

(24) $$\begin{array}{cc} \pi_{\zeta }\left(\zeta |\eta ,x\right) & \propto \pi_{2}\left(e^{\zeta }|e^{\eta },x\right)\cdot e^{\zeta }. \end{array}$$

Given the current state $\left(\eta^{\left(l-1\right)},\zeta^{\left(l-1\right)}\right)$, we update η and ζ sequentially via Metropolis–Hastings steps using Gaussian random-walk proposal distributions. At the end of each iteration, the updated parameters are transformed back to their original positive space via the inverse mapping $\alpha^{\left(l\right)}=\exp\left(\eta^{\left(l\right)}\right)$ and $\beta^{\left(l\right)}=\exp\left(\zeta^{\left(l\right)}\right)$. This yields a Markov chain whose stationary distribution corresponds to the joint posterior of the original scale and shape parameters. The detailed procedure is summarized in Algorithm 2.

Upon completion, Algorithm 2 yields a sequence of draws ${\left\{\left(\alpha^{\left(l\right)},\beta^{\left(l\right)}\right)\right\}}_{l=1}^{N}$. To mitigate the influence of initial values and allow the Markov chains to approach the stationary distribution, the first *B* iterations are discarded as burn-in. Furthermore, to reduce sample autocorrelation, a thinning interval of *h* is applied to the post-burn-in chains. This yields an effective sample of size M=(N−B)/h.

For notational convenience, let $l^{\prime }\in \left\{1,2,\ldots ,M\right\}$ denote the index of these final retained draws, defining the re-indexed sequence as ${\left\{\left(\alpha^{\left(l^{\prime }\right)},\beta^{\left(l^{\prime }\right)}\right)\right\}}_{l^{\prime }=1}^{M}$. Assuming adequate MCMC convergence, these samples are treated as approximately independent realizations from the joint posterior distribution $\pi_{jp}\left(\alpha ,\beta |x\right)$. Consequently, Bayesian inference proceeds via standard Monte Carlo integration.

For any parametric function of interest θ=g(α,β), its Bayes estimator under the SEL is approximated by the empirical average:

(25) $${\hat{\theta }}_{BE}\approx \frac{1}{M}\sum_{l^{\prime }=1}^{M}g\left(\alpha^{\left(l^{\prime }\right)},\beta^{\left(l^{\prime }\right)}\right).$$

Similarly, the posterior uncertainty of θ is characterized by a 100(1−ξ)% equal-tailed credible interval. By evaluating the function for each retained sample, we obtain a sequence of posterior realizations $\theta_{l^{\prime }}=g\left(\alpha^{\left(l^{\prime }\right)},\beta^{\left(l^{\prime }\right)}\right)$ for $l^{\prime }=1,\ldots ,M$. Sorting these values in ascending order yields the order statistics $\theta_{\left(1\right)}\leq \theta_{\left(2\right)}\leq \cdots \leq \theta_{\left(M\right)}$. The approximate 100(1−ξ)% credible interval is then constructed directly from the empirical quantiles:

(26) $$\left(\theta_{\left(\lfloor M\xi /2\rfloor \right)},\;\theta_{\left(\lfloor M(1-\xi /2)\rfloor \right)}\right),$$

where ⌊·⌋ denotes the floor function. Setting g(α,β)=α or β directly provides the respective Bayes estimates and credible intervals for the individual parameters.
**Algorithm 2** Metropolis–Hastings-within-Gibbs sampler for (α,β)  1:**Input:** Progressively censored data x, total iterations *N*, burn-in length *B*, proposal standard deviations $s_{\eta }$ and $s_{\zeta }$.  2:**Initialization:** Set initial values $\eta^{\left(0\right)}$ and $\zeta^{\left(0\right)}$ (equivalently, $\alpha^{\left(0\right)}=\exp\left(\eta^{\left(0\right)}\right)$ and $\beta^{\left(0\right)}=\exp\left(\zeta^{\left(0\right)}\right)$).  3:**For** l=1,2,…,N **do**:  4:   **Update η:**  5:       Generate a candidate $\eta^{*}∼N\;\left(\eta^{\left(l-1\right)},\;s_{\eta }^{2}\right)$.  6:       Compute the acceptance ratio using the log-space kernel (23):$$R_{\eta }=\min\left\{1,\;\frac{\pi_{\eta }\;\left(\eta^{*}|\zeta^{\left(l-1\right)},x\right)}{\pi_{\eta }\;\left(\eta^{\left(l-1\right)}|\zeta^{\left(l-1\right)},x\right)}\right\}.$$  7:       Generate $u_{\eta }∼\mathrm{Uniform}(0,1)$.  8:       **If** $u_{\eta }\leq R_{\eta }$ **then** $\eta^{\left(l\right)}=\eta^{*}$ **else** $\eta^{\left(l\right)}=\eta^{\left(l-1\right)}$.  9:   **Update ζ:**10:      Generate a candidate $\zeta^{*}∼N\;\left(\zeta^{\left(l-1\right)},\;s_{\zeta }^{2}\right)$.11:      Compute the acceptance ratio using the log-space kernel (24):$$R_{\zeta }=\min\left\{1,\;\frac{\pi_{\zeta }\;\left(\zeta^{*}|\eta^{\left(l\right)},x\right)}{\pi_{\zeta }\;\left(\zeta^{\left(l-1\right)}|\eta^{\left(l\right)},x\right)}\right\}.$$12:      Generate $u_{\zeta }∼\mathrm{Uniform}(0,1)$.13:      **If** $u_{\zeta }\leq R_{\zeta }$ **then** $\zeta^{\left(l\right)}=\zeta^{*}$ **else** $\zeta^{\left(l\right)}=\zeta^{\left(l-1\right)}$.14:   **Parameter Transformation:** $\alpha^{\left(l\right)}=\exp\;\left(\eta^{\left(l\right)}\right)$ and $\beta^{\left(l\right)}=\exp\;\left(\zeta^{\left(l\right)}\right)$.15:**End For**16:**Output:** The joint posterior MCMC sequence ${\left\{\left(\alpha^{\left(l\right)},\beta^{\left(l\right)}\right)\right\}}_{l=1}^{N}$.

## 4. Point Predictor

Predicting the future failure times of censored units is a pivotal topic in reliability analysis. In this section, we develop both frequentist and Bayesian frameworks to predict these unobserved lifetimes based on the informative sample x. Specifically, our objective is to predict the *k*-th order statistic, $Y=Y_{k:r_{j}}$ ($1\leq k\leq r_{j}$), from the set of $r_{j}$ surviving units removed at the *j*-th censoring stage.

### 4.1. Best Unbiased Predictor

In the frequentist framework, the Best Unbiased Predictor (BUP) of the future statistic *Y*, denoted as $Y_{BUP}^{*}$, is defined as the estimator that minimizes the Mean Squared Prediction Error (MSPE), $E[{\left(Y^{*}-Y\right)}^{2}]$, subject to the unbiasedness constraint $E[Y^{*}-Y]=0$. A fundamental result in statistical prediction theory establishes that the BUP is equivalent to the conditional expectation of *Y* given the observed censored sample x. Consequently, the BUP can be expressed as

(27) $$Y_{BUP}^{*}\left(x\right)=E\left[Y|x\right]=\int_{x_{j}}^{\infty }y\cdot f_{Y|x}\left(y|\alpha ,\beta \right)\;dy,$$

where $f_{Y|x}\left(\cdot |\alpha ,\beta \right)$ represents the conditional probability density function (PDF) of the future observation given the model parameters.

To evaluate the integral in (27), we must first derive the explicit form of the conditional density $f_{Y|x}$. This derivation relies on the Markovian property of order statistics (see David and Nagaraja [30]). This property implies that, given the observed progressive censoring sequence x, the probabilistic behavior of the future failure times removed at stage *j* depends only on the immediate failure time $x_{j}$ at which they were censored, independent of the earlier failure times $x_{1},\ldots ,x_{j-1}$.

Specifically, the conditional distribution of *Y* is equivalent to the distribution of the *k*-th order statistic from a sample of size $r_{j}$ drawn from the parent distribution truncated on the left by $x_{j}$. The cumulative distribution function (CDF) of this truncated distribution is given by $G_{j}\left(y\right)=\left[F\left(y\right)-F\left(x_{j}\right)\right]/S\left(x_{j}\right)$ for $y>x_{j}$. Consequently, the conditional PDF of *Y* is derived as

(28) fY|x(y|α,β)=Ck,rj∑v=0k−1k−1v(−1)k−1−v1−F(y)1−F(xj)rj−vf(y)1−F(y),

where $C_{k,r_{j}}=\frac{r_{j}!}{\left(k-1\right)!\left(r_{j}-k\right)!}$ is the normalizing constant. By substituting the PDF and CDF of the Log-Logistic distribution from Equations (1)–(3) into (28), we obtain the explicit form of the conditional density for the LLD:

(29) $$f_{Y|x}\left(y|\alpha ,\beta \right)=C_{k,r_{j}}\sum_{v=0}^{k-1}W_{v}{\left[\frac{1+{\left(x_{j}/\alpha \right)}^{\beta }}{1+{\left(y/\alpha \right)}^{\beta }}\right]}^{r_{j}-v}\frac{\beta }{\alpha }\frac{{\left(y/\alpha \right)}^{\beta -1}}{1+{\left(y/\alpha \right)}^{\beta }},$$

where Wv=k−1v(−1)k−1−v.

Substituting the explicit conditional density derived in (29) into the expectation integral (27), the BUP can be expressed as a weighted sum of integrals. However, direct numerical evaluation of these integrals is computationally unstable due to the infinite upper integration limit $\left(x_{j},\infty \right)$. To circumvent this, we apply the variable transformation $t=\frac{y-x_{j}}{1+y-x_{j}}$, which maps the semi-infinite domain to the finite unit interval (0,1). The inverse transformation is $y=x_{j}+\frac{t}{1-t}$, with the Jacobian $dy=\frac{1}{{\left(1-t\right)}^{2}}dt$.

Consequently, the conditional expectation takes the following computational form:

(30) $$E\left[Y|x,\alpha ,\beta \right]=C_{k,r_{j}}\sum_{v=0}^{k-1}W_{v}{\left[1+{\left(\frac{x_{j}}{\alpha }\right)}^{\beta }\right]}^{r_{j}-v}\times J_{v}\left(x_{j},\alpha ,\beta \right),$$

where the integral component $J_{v}$ is defined over the unit interval as

(31) $$\begin{array}{cc} J_{v}\left(x_{j},\alpha ,\beta \right) & =\int_{0}^{1}\left(x_{j}+\frac{t}{1-t}\right)\frac{\beta }{\alpha }{\left(\frac{x_{j}+\frac{t}{1-t}}{\alpha }\right)}^{\beta -1}\\ \; & \;\times \;{\left[1+{\left(\frac{x_{j}+\frac{t}{1-t}}{\alpha }\right)}^{\beta }\right]}^{-(r_{j}-v+1)}\frac{1}{{\left(1-t\right)}^{2}}\;dt. \end{array}$$

Under the squared error loss, the BUP is optimal in the sense that its conditional prediction risk is exactly the conditional variance of *Y*, namely,

(32) $$E\left[{\left(Y-Y_{BUP}^{*}\left(x\right)\right)}^{2}\;|\;x,\alpha ,\beta \right]=\mathrm{Var}\left(Y|x,\alpha ,\beta \right).$$

Moreover,

(33) $$\begin{array}{cc} \mathrm{Var}(Y|x,\alpha ,\beta ) & =E\left[Y^{2}|x,\alpha ,\beta \right]-{\left(E[Y|x,\alpha ,\beta ]\right)}^{2}\\ \; & =C_{k,r_{j}}\sum_{v=0}^{k-1}W_{v}{\left[1+{\left(\frac{x_{j}}{\alpha }\right)}^{\beta }\right]}^{r_{j}-v}J_{v}^{\left(2\right)}\left(x_{j},\alpha ,\beta \right)-{\left(E[Y|x,\alpha ,\beta ]\right)}^{2}, \end{array}$$

where

(34) $$\begin{array}{cc} J_{v}^{\left(2\right)}\left(x_{j},\alpha ,\beta \right)\; & =\int_{0}^{1}{\left(x_{j}+\frac{t}{1-t}\right)}^{2}\frac{\beta }{\alpha }{\left(\frac{x_{j}+\frac{t}{1-t}}{\alpha }\right)}^{\beta -1}\\ \; & \;\times {\left[1+{\left(\frac{x_{j}+\frac{t}{1-t}}{\alpha }\right)}^{\beta }\right]}^{-(r_{j}-v+1)}\frac{1}{{\left(1-t\right)}^{2}}\;dt. \end{array}$$

Thus, the conditional variance can be evaluated numerically in the same manner as the conditional mean, with the integrand modified from $\left(x_{j}+\frac{t}{1-t}\right)$ to ${\left(x_{j}+\frac{t}{1-t}\right)}^{2}$.

In practice, since the true parameters (α,β) are unknown, the theoretical predictor $Y_{BUP}^{*}$ cannot be computed directly. Instead, the estimated BUP, denoted as ${\hat{Y}}_{BUP}$, is obtained by substituting the Maximum Likelihood Estimators (MLEs), ${\hat{\alpha }}_{MLE}$ and ${\hat{\beta }}_{MLE}$, into the expectation kernel derived above:

(35) $${\hat{Y}}_{BUP}=E\left[Y|x,{\hat{\alpha }}_{MLE},{\hat{\beta }}_{MLE}\right].$$

The integral $J_{v}$ in (31) is then evaluated using standard numerical quadrature methods (e.g., the Romberg method) at these point estimates.

This plug-in construction introduces additional variability through parameter estimation. By the asymptotic normality of the MLE and a first-order delta-method argument, the estimation-induced variability of ${\hat{Y}}_{BUP}=T\left(\hat{\phi }\right)$ can be approximated by

(36) $$\hat{\mathrm{Var}}\left({\hat{Y}}_{BUP}\right)\approx \nabla T{\left(\hat{\phi }\right)}^{⊤}J_{n}^{-1}\left(\hat{\phi }\right)\nabla T\left(\hat{\phi }\right),$$

where T(ϕ)=E[Y∣x,ϕ], and $\nabla T(\hat{\phi })$ denotes the gradient of *T* evaluated at $\hat{\phi }$. In the simulation study, the reported Mean Squared Prediction Error (MSPE) can therefore be viewed as a Monte Carlo approximation to the prediction risk of the BUP under squared error loss.

### 4.2. Conditional Median Predictor

As discussed previously, when the shape parameter of the Log-Logistic distribution approaches the infinite-variance boundary (β≤2), the conditional expectation E[Y|x] may become numerically unstable. To obtain a more stable point predictor in such heavy-tailed settings, we consider the Conditional Median Predictor (CMP).

The CMP of *Y*, denoted as $Y_{CMP}^{*}$, is formally defined as the median of the conditional distribution of *Y* given the observed data x. To obtain an explicit closed-form expression for $Y_{CMP}^{*}$, we leverage the truncated distribution G(·) established in Section 4.1.

According to the probability integral transform for order statistics, evaluating the truncated CDF $G_{j}\left(\cdot \right)$ at the future failure time *Y* yields a new random variable *U* that follows a Beta distribution. That is,

(37) $$U=G\left(Y\right)∼\mathrm{Beta}(k,r_{j}-k+1).$$

Consequently, the median of the conditional distribution of *Y* corresponds to the median of this Beta distribution. Let $m_{k,r_{j}}$ denote the median of the $\mathrm{Beta}(k,r_{j}-k+1)$ distribution, which can be readily found by solving the regularized incomplete beta function $I_{m_{k,r_{j}}}\left(k,r_{j}-k+1\right)=0.5$. It naturally follows that $Y_{CMP}^{*}$ must satisfy

(38) $$G_{j}\left(Y_{CMP}^{*}\right)=\frac{F\left(Y_{CMP}^{*}\right)-F\left(x_{j}\right)}{S(x_{j})}=m_{k,r_{j}}.$$

Rearranging (38) in terms of the survival function S(·), we obtain a relationship:

(39) $$S\left(Y_{CMP}^{*}\right)=\left(1-m_{k,r_{j}}\right)S\left(x_{j}\right).$$

Next, by directly substituting the explicit survival function of the Log-Logistic distribution from Equation (3) into (39), the equation expands to

(40) $$\frac{1}{1+{\left(Y_{CMP}^{*}/\alpha \right)}^{\beta }}=\frac{1-m_{k,r_{j}}}{1+{\left(x_{j}/\alpha \right)}^{\beta }}.$$

Solving this algebraic equation for $Y_{CMP}^{*}$ yields the explicit theoretical formulation for the Conditional Median Predictor:

(41) $$Y_{CMP}^{*}\left(\alpha ,\beta \right)=\alpha {\left[\frac{m_{k,r_{j}}+{\left(x_{j}/\alpha \right)}^{\beta }}{1-m_{k,r_{j}}}\right]}^{\frac{1}{\beta }}.$$

In practice, since the true parameters are unknown, the estimated Conditional Median Predictor is obtained by plugging the MLEs into Equation (41):

(42) $${\hat{Y}}_{CMP}={\hat{\alpha }}_{MLE}{\left[\frac{m_{k,r_{j}}+{\left(x_{j}/{\hat{\alpha }}_{MLE}\right)}^{{\hat{\beta }}_{MLE}}}{1-m_{k,r_{j}}}\right]}^{\frac{1}{{\hat{\beta }}_{MLE}}}.$$

The predictor in (42) is computationally attractive because it admits a closed-form expression and avoids the unstable conditional expectations that may arise in heavy-tailed settings.

Its variability comes from two sources. The first is the conditional randomness of the future order statistic *Y* itself. The second is parameter estimation, since the practical predictor ${\hat{Y}}_{CMP}$ is obtained by replacing the unknown parameters with their MLEs. Hence, the plug-in predictor inherits additional uncertainty through the estimation errors of ${\hat{\alpha }}_{MLE}$ and ${\hat{\beta }}_{MLE}$. In the simulation study, MSPE is used as a common criterion to evaluate and compare the prediction performance of all competing methods.

### 4.3. Bayesian Predictor

Based on the definition of the posterior predictive density, the predictive density of *Y* given the observed data x is expressed as

(43) $$p\left(y|x\right)=\int_{0}^{\infty }\int_{0}^{\infty }f_{Y|x}\left(y|\alpha ,\beta \right)\;\pi \left(\alpha ,\beta |x\right)\;d\alpha \;d\beta ,\;y>x_{j},$$

where π(α,β|x) is the joint posterior density derived in the previous section.

Substituting (29) into (43), the posterior predictive density becomes

(44) $$\begin{array}{cc} p(y|x) & =C_{k,r_{j}}\int_{0}^{\infty }\int_{0}^{\infty }\sum_{v=0}^{k-1}W_{v}{\left[1+{\left(\frac{x_{j}}{\alpha }\right)}^{\beta }\right]}^{r_{j}-v}\\ \; & \;\times \;\frac{\beta }{\alpha }{\left(\frac{y}{\alpha }\right)}^{\beta -1}{\left[1+{\left(\frac{y}{\alpha }\right)}^{\beta }\right]}^{-(r_{j}-v+1)}\pi \left(\alpha ,\beta |x\right)\;d\alpha \;d\beta . \end{array}$$

Under the Squared Error Loss (SEL) function, the Bayesian Predictor (BP) of $Y=Y_{k:r_{j}}$ is the mean of the posterior predictive distribution and is defined as

(45) $$\begin{array}{cc} Y_{BP}^{*}=E\left[Y|x\right] & =\int_{x_{j}}^{\infty }y\cdot p\left(y|x\right)\;dy\\ \; & =\int_{0}^{\infty }\int_{0}^{\infty }\left(\int_{x_{j}}^{\infty }y\cdot f_{Y|x}\left(y|\alpha ,\beta \right)\;dy\right)\pi \left(\alpha ,\beta |x\right)\;d\alpha \;d\beta \\ \; & =\int_{0}^{\infty }\int_{0}^{\infty }E\left[Y|x,\alpha ,\beta \right]\;\pi \left(\alpha ,\beta |x\right)\;d\alpha \;d\beta . \end{array}$$

Equation (45) also serves as the basis for quantifying the posterior uncertainty of the Bayesian Predictor. Under the Squared Error Loss (SEL) function, the posterior prediction risk is determined by the posterior predictive distribution of *Y* given x and reduces to the posterior predictive variance:

(46) $$E\left[{\left(Y-Y_{BP}^{*}\right)}^{2}\;|\;x\right]=\mathrm{Var}\left(Y|x\right).$$

Furthermore, by the law of total variance,

(47) $$\mathrm{Var}\left(Y|x\right)=E_{\pi }\;\left[\mathrm{Var}(Y|x,\alpha ,\beta )\;|\;x\right]+{\mathrm{Var}}_{\pi }\;\left(E[Y|x,\alpha ,\beta ]\;|\;x\right),$$

where the first term reflects the average conditional variability of the future observation given the model parameters, and the second term captures the additional uncertainty induced by posterior variation in (α,β).

Since neither the posterior predictive mean in (45) nor the variance components in (47) admit closed-form evaluation in general, we approximate them using the retained MCMC samples ${\left\{\left(\alpha^{\left(l^{\prime }\right)},\beta^{\left(l^{\prime }\right)}\right)\right\}}_{l^{\prime }=1}^{M}$ generated from the posterior distribution. Using these retained samples and the computational form established in Equations (30) and (31), the simulation-consistent Bayesian Predictor is given by

(48) $$\begin{array}{cc} {\hat{Y}}_{BP} & =\frac{1}{M}\sum_{l^{\prime }=1}^{M}E\left[Y|x,\alpha^{\left(l^{\prime }\right)},\beta^{\left(l^{\prime }\right)}\right]\\ \; & =\frac{C_{k,r_{j}}}{M}\sum_{l^{\prime }=1}^{M}\sum_{v=0}^{k-1}W_{v}{\left[1+{\left(\frac{x_{j}}{\alpha^{\left(l^{\prime }\right)}}\right)}^{\beta^{\left(l^{\prime }\right)}}\right]}^{r_{j}-v}J_{v}\left(x_{j},\alpha^{\left(l^{\prime }\right)},\beta^{\left(l^{\prime }\right)}\right). \end{array}$$

For each MCMC iteration, the integral $J_{v}$ is computed numerically as described in Section 4.1.

Correspondingly, let

$$\mu^{\left(l^{\prime }\right)}=E\left[Y|x,\alpha^{\left(l^{\prime }\right)},\beta^{\left(l^{\prime }\right)}\right],\;\sigma^{2(l^{\prime })}=\mathrm{Var}\left(Y|x,\alpha^{\left(l^{\prime }\right)},\beta^{\left(l^{\prime }\right)}\right).$$

Then, a Monte Carlo approximation to the posterior predictive variance is

(49) $$\hat{\mathrm{Var}}\left(Y|x\right)=\frac{1}{M}\sum_{l^{\prime }=1}^{M}\sigma^{2(l^{\prime })}+\frac{1}{M-1}\sum_{l^{\prime }=1}^{M}{\left(\mu^{\left(l^{\prime }\right)}-\bar{\mu }\right)}^{2},$$

where

$$\bar{\mu }=\frac{1}{M}\sum_{l^{\prime }=1}^{M}\mu^{\left(l^{\prime }\right)}.$$

Here, each $\mu^{\left(l^{\prime }\right)}$ is computed from (48), while each $\sigma^{2(l^{\prime })}$ is obtained from the conditional variance expression developed in Section 4.1. To evaluate the long-run predictive performance of the proposed methods, the subsequent simulation study relies on the empirical MSPE computed across repeated Monte Carlo samples.

## 5. Prediction Interval

In this section, we derive prediction intervals (PIs) for the future failure time $Y=Y_{k:r_{j}}$. Both frequentist and Bayesian approaches rely on the conditional survival function of *Y* given the observed data x, denoted as $S_{Y|x}\left(y|\alpha ,\beta \right)=P\left(Y>y|x,\alpha ,\beta \right)$.

Based on the conditional PDF derived in Equation (29), the explicit form of the conditional survival function for the Log-Logistic distribution is obtained by integration:

(50) $$S_{Y|x}\left(y|\alpha ,\beta \right)=\int_{y}^{\infty }f_{Y|x}\left(z|\alpha ,\beta \right)\;dz=C_{k,r_{j}}\sum_{v=0}^{k-1}\frac{W_{v}}{r_{j}-v}{\left[\frac{1+{\left(x_{j}/\alpha \right)}^{\beta }}{1+{\left(y/\alpha \right)}^{\beta }}\right]}^{r_{j}-v},\;y>x_{j}.$$

### 5.1. Pivotal Prediction Intervals

To construct a frequentist prediction interval, we employ the plug-in pivotal method. Although the distribution of $S_{Y|x}\left(Y\right)$ is parameter-free (pivotal), the true parameters are unknown. We therefore approximate the survival function by substituting the MLEs, $\hat{\alpha }$ and $\hat{\beta }$, directly into Equation (50).

The approximate 100(1−γ)% equal-tailed frequentist prediction interval, $\left(L_{F},U_{F}\right)$, is obtained by solving the following nonlinear equations:

(51) $$S_{Y|x}\left(L_{F}|\hat{\alpha },\hat{\beta }\right)=1-\frac{\gamma }{2}\;\mathrm{and}\;S_{Y|x}\left(U_{F}|\hat{\alpha },\hat{\beta }\right)=\frac{\gamma }{2}.$$

Since $S_{Y|x}\left(y|\hat{\alpha },\hat{\beta }\right)$ is a strictly decreasing continuous function of *y* in the domain $\left(x_{j},\infty \right)$, the roots $L_{F}$ and $U_{F}$ are unique and can be determined efficiently using numerical root-finding algorithms such as Brent’s method.

### 5.2. Bayesian Prediction Intervals (BPI)

In the Bayesian framework, we account for parameter uncertainty by constructing the posterior predictive survival function, $S_{P}\left(y|x\right)$. This is defined as the expectation of the conditional survival function over the posterior distribution:

(52) $$S_{P}\left(y|x\right)=\int_{0}^{\infty }\int_{0}^{\infty }S_{Y|x}\left(y|\alpha ,\beta \right)\;\pi \left(\alpha ,\beta |x\right)\;d\alpha \;d\beta .$$

Using the retained MCMC samples ${\left\{\left(\alpha^{\left(l^{\prime }\right)},\beta^{\left(l^{\prime }\right)}\right)\right\}}_{l^{\prime }=1}^{M}$ generated from the posterior, the simulation-consistent estimator of the predictive survival function is

(53) $${\hat{S}}_{P}\left(y|x\right)=\frac{1}{M}\sum_{l^{\prime }=1}^{M}S_{Y|x}\left(y|\alpha^{\left(l^{\prime }\right)},\beta^{\left(l^{\prime }\right)}\right)=\frac{C_{k,r_{j}}}{M}\sum_{l^{\prime }=1}^{M}\sum_{i=0}^{k-1}\frac{W_{i}}{r_{j}-i}{\left[\frac{1+{\left(x_{j}/\alpha^{\left(l^{\prime }\right)}\right)}^{\beta^{\left(l^{\prime }\right)}}}{1+{\left(y/\alpha^{\left(l^{\prime }\right)}\right)}^{\beta^{\left(l^{\prime }\right)}}}\right]}^{r_{j}-i}.$$

The 100(1−γ)% equal-tailed Bayesian Prediction interval, $\left(L_{B},U_{B}\right)$, is then obtained by solving

(54) $${\hat{S}}_{P}\left(L_{B}|x\right)=1-\frac{\gamma }{2}\;\mathrm{and}\;{\hat{S}}_{P}\left(U_{B}|x\right)=\frac{\gamma }{2}.$$

### 5.3. Highest Posterior Density (HPD) Prediction Intervals

While the equal-tailed Bayesian Prediction interval is straightforward to implement, it is generally suboptimal for asymmetric models. Because the Log-Logistic distribution exhibits significant right-skewness, particularly under heavy-tailed regimes (β≤2), the equal-tailed approach symmetrically trims probability mass, thereby extending the upper bound far into the flat right tail. To achieve a more precise uncertainty quantification, we construct the Highest Posterior Density (HPD) prediction interval, which minimizes the interval width for a designated credible level.

Theoretically, the exact 100(1−γ)% HPD prediction interval, denoted as $\left(L_{HPD},U_{HPD}\right)$, must satisfy two constraints: enforcing the nominal coverage probability and ensuring identical boundary densities. This is mathematically expressed by the following system of non-linear equations:

(55) $$\left\{\begin{array}{c} \int_{L_{HPD}}^{U_{HPD}}p\left(y|x\right)\;dy=1-\gamma ,\\ p\left(L_{HPD}|x\right)=p\left(U_{HPD}|x\right). \end{array}\right.$$

Directly solving this system is analytically intractable. Substituting the complex posterior predictive density p(y|x) into (55) results in a set of equations involving double integrals and the non-standard joint posterior. Consequently, a closed-form solution for $L_{HPD}$ and $U_{HPD}$ is unavailable.

To address this computationally, we employ a simulation-based empirical procedure following the framework of Chen and Shao [31]. Using the previously retained MCMC samples ${\left\{\left(\alpha^{\left(l^{\prime }\right)},\beta^{\left(l^{\prime }\right)}\right)\right\}}_{l^{\prime }=1}^{M}$, we simulate a future observation $y^{\left(l^{\prime }\right)}$ from the conditional predictive distribution for each iteration $l^{\prime }=1,2,\ldots ,M$. By applying the inverse transform sampling method, $y^{\left(l^{\prime }\right)}$ is generated by equating the conditional survival function to a uniform random variable u∼Uniform(0,1):

(56) $$S_{Y|x}\left(y^{\left(l^{\prime }\right)}|\alpha^{\left(l^{\prime }\right)},\beta^{\left(l^{\prime }\right)}\right)=u.$$

Substituting the explicit formulation of the conditional survival function, Equation (56) expands to

(57) $$C_{k,r_{j}}\sum_{v=0}^{k-1}\frac{W_{v}}{r_{j}-v}{\left[\frac{1+{\left(x_{j}/\alpha^{\left(l^{\prime }\right)}\right)}^{\beta^{\left(l^{\prime }\right)}}}{1+{\left(y^{\left(l^{\prime }\right)}/\alpha^{\left(l^{\prime }\right)}\right)}^{\beta^{\left(l^{\prime }\right)}}}\right]}^{r_{j}-v}=u.$$

Since Equation (57) does not admit a closed-form inverse, the predictive root $y^{\left(l^{\prime }\right)}$ is obtained numerically via Brent’s method at each MCMC iteration.

The resulting sequence $\left\{y^{\left(1\right)},y^{\left(2\right)},\ldots ,y^{\left(M\right)}\right\}$ forms a random sample from the posterior predictive density p(y|x). By sorting these simulated predictions in ascending order such that $y_{\left(1\right)}\leq y_{\left(2\right)}\leq \cdots \leq y_{\left(M\right)}$, the integral system in Equation (55) is reduced to a discrete search problem. The 100(1−γ)% empirical HPD prediction interval is constructed by identifying the shortest contiguous window containing exactly ⌊(1−γ)M⌋+1 samples. Let $w^{*}$ be the starting index that minimizes the interval width:

(58) $$w^{*}=\underset{1\leq w\leq M-\lfloor (1-\gamma )M\rfloor }{\mathrm{argmin}}\left(y_{\left(w+\lfloor (1-\gamma )M\rfloor \right)}-y_{\left(w\right)}\right).$$

The approximate HPD prediction interval is directly given by $\left(y_{\left(w^{*}\right)},\;y_{\left(w^{*}+\left\lfloor \left(1-\gamma \right)M\right\rfloor \right)}\right)$. This data-driven approach effectively circumvents the analytical intractability while preserving the optimal width property of the HPD interval.

## 6. Simulation Study

In this section, we conduct a Monte Carlo simulation study to evaluate the finite-sample performance of the proposed point predictors (BUP, CMP, BP) and interval predictors (PP, BPI, HPD).

As a baseline configuration, we generate progressive Type-II censored samples with n=75 and m=50 so that n−m=25 units are removed in total, corresponding to a censoring proportion of 33.3%. This configuration reflects a typical reliability testing scenario, balancing experimental duration with the statistical efficiency required for reliable estimation.

To further examine the effects of sample size and censoring proportion, we additionally conduct two sensitivity analyses. Specifically, we first consider proportional (n,m) settings with fixed m/n=2/3, namely (60,40), (75,50), (90,60), and (120,80), to study the effect of increasing sample size under a stable censoring proportion. We then fix n=75 and vary *m* over {30,40,50,60}, corresponding to m/n∈{0.400,0.533,0.667,0.800}, to study the effect of increasing the observed-failure proportion under a fixed total sample size.

Following Abou Ghaida and Baklizi [32], the true scale parameter is set to α=3.0. Because α acts purely as a scale parameter, its specific value merely scales the time axis without altering the underlying distribution shape. Therefore, fixing α entails no loss of generality, as the relative performance of the prediction methods remains theoretically unaffected.

The shape parameter is varied as β∈{5.0,2.0,1.5}. For the Log-Logistic (Fisk) distribution, the *r*-th raw moment exists if and only if β>r; in particular, the mean exists for β>1 and the variance exists for β>2 [33,34,35].

- β=5.0: A finite-variance regime (β>2) where both the mean and variance exist. This serves as a baseline scenario where classical expectation-based predictors typically perform well.
- β=2.0: The infinite-variance boundary, marking the critical transition to pronounced heavy-tailed behavior.
- β=1.5: Astrict heavy-tailed regime (1<β<2) where the mean exists but the variance is infinite. In this regime, conventional predictors relying on the $L_{2}$ norm are prone to severe error inflation [36].

This parameter configuration enables a systematic comparison of predictive performance across progressively heavier tails. As β decreases toward and below 2, extreme realizations occur more frequently, so performance summaries driven by squared deviations become more sensitive to large errors. Examining the competing methods under these three regimes helps reveal how their point and interval predictions respond to increasing tail heaviness.

Importantly, from a theoretical perspective, the nonexistence of higher-order raw moments of the Log-Logistic distribution in the heavier-tailed regimes does not, by itself, invalidate likelihood-based inference for the model parameters (α,β). The convergence behavior of the MLE and Bayes estimators is governed by the censored likelihood structure, parameter identifiability, and the effective information contained in the progressively censored sample, rather than by the existence of all raw moments of the lifetime variable itself. What changes in the heavy-tailed settings is mainly the finite-sample stability: as β approaches or falls below the variance boundary, extreme realizations become more frequent, squared-error-based evaluation criteria become inherently less stable, and the empirical convergence of error summaries tends to be slower. Therefore, although parameter estimation remains theoretically justified and meaningful in these regimes, one should expect larger variability and slower finite-sample improvement than in the light-tailed case.

Three structurally distinct progressive censoring schemes are evaluated:

- Scheme I (Early Censoring): R=(25,0,…,0). All removals occur at the first observed failure. In engineering practice, this mimics burn-in screening or facility reallocation scenarios, where test channels are freed up for subsequent batches after capturing initial defective units (infant mortalities).
- Scheme II (Late Censoring): R=(0,…,0,25). All removals are delayed until the terminal *m*-th failure, matching conventional Type-II right censoring. This corresponds to standard life testing setups where components are continuously monitored without intermediate interruption until the required failure quota is met, terminating the experiment to save long-term operational costs.
- Scheme III (Uniform Censoring): R=(1,0,1,0,…,1,0). Removals are distributed evenly throughout the testing process. This design is highly relevant to scheduled periodic maintenance or destructive post-mortem inspections, where surviving components are systematically withdrawn at various life stages to analyze internal degradation and wear mechanisms.

To evaluate the predictive robustness across different extrapolation horizons and conditional variances, we specify three representative targets for the *k*-th future failure among the $r_{j}$ units withdrawn at stage *j*:

- Target 1 (Early-Min): j=1,k=1. This target represents the first failure among units withdrawn at the initial stage. It serves as a short-horizon prediction where the temporal gap between the last observation and the target is minimal.
- Target 2 (Early-Median): $j=1,k=\lfloor r_{1}/2\rfloor$. This involves predicting the median failure time among the initially removed units. Compared to Target 1, it requires a more extensive forward extrapolation, testing the model’s ability to capture the heavy-tailed behavior of the Log-Logistic distribution.
- Target 3 (Late-Min): j=m,k=1. This target focuses on the first failure among units removed at the terminal stage. It is designed for deep-tail prediction, assessing the accuracy of the predictors at the very end of the experimental life cycle.

For the Bayesian framework, joint posterior samples are generated using a Metropolis–Hastings within Gibbs algorithm in the unconstrained log-space. For each simulated dataset, the algorithm is run for *N* = 10,000 iterations. To mitigate the influence of initial values and allow the Markov chains to approach the stationary distribution, the first *B* = 2000 iterations are discarded as burn-in. The adequacy of this burn-in period was verified during pilot runs by examining trace plots for stationarity and autocorrelation function (ACF) plots for mixing behavior.

During the automated Monte Carlo replications, the proposal variances are dynamically tuned to maintain the M-H acceptance rates within the theoretically optimal range of 20% to 50%, promoting adequate exploration of the parameter space. To reduce sample autocorrelation, a thinning interval of 10 is applied to the post-burn-in chains, yielding a final sample of M=800 roughly independent draws for subsequent posterior inference.

Bayesian inference is conducted under two distinct prior specifications:

- Prior 0 (Non-informative): The standard Jeffreys prior $\pi \left(\alpha ,\beta \right)\propto {\left(\alpha \beta \right)}^{-1}$. Although strictly an improper prior, it mathematically transforms into a flat uniform prior (π(η,ζ)∝1) in the unconstrained log-space (η=lnα,ζ=lnβ) after multiplying by the Jacobian $\left|J\right|=e^{\eta }e^{\zeta }$. Consequently, the undefined proportionality constant cancels out within the Metropolis–Hastings acceptance ratio, reducing the target density ratio directly to the likelihood ratio. To ensure numerical stability and prevent the Markov chains from drifting into pathological regions during computation, this prior is practically implemented with a mild truncation ($\alpha >10^{-4}$ and β>1.05). This guarantees algorithmic validity provided the posterior is proper (i.e., m≥2).
- Prior 1 (Empirical Bayes): Independent Gamma priors $\alpha ∼\mathrm{Gamma}(a_{\alpha },b_{\alpha })$ and $\beta ∼\mathrm{Gamma}(a_{\beta },b_{\beta })$, where the hyperparameters are dynamically calibrated by the MLEs of the current sample. We set $a_{\alpha }=a_{\beta }=30$, $b_{\alpha }=30/{\hat{\alpha }}_{MLE}$, and $b_{\beta }=30/{\hat{\beta }}_{MLE}$. This configuration yields a prior coefficient of variation of approximately 18% ($1/\sqrt{30}$), providing sufficient empirical regularization in heavy-tailed regimes without overly dominating the likelihood.

The performance of the predictors is evaluated over $N_{rep}$ = 1000 independent Monte Carlo replications. Let ${\hat{Y}}^{\left(s\right)}$ and $Y_{obs}^{\left(s\right)}$ denote the predicted value and the true realized future failure time in the *s*-th replication, respectively.

For point predictors, the accuracy is quantified using Bias and Mean Squared Prediction Error (MSPE):

(59) $$\mathrm{Bias}=\frac{1}{N_{rep}}\sum_{s=1}^{N_{rep}}\left({\hat{Y}}^{\left(s\right)}-Y_{obs}^{\left(s\right)}\right),\;\mathrm{MSPE}=\frac{1}{N_{rep}}\sum_{s=1}^{N_{rep}}{\left({\hat{Y}}^{\left(s\right)}-Y_{obs}^{\left(s\right)}\right)}^{2}.$$

For interval predictors (PP, BPI, and HPD) with a nominal confidence level of 1−γ=0.95, the performance is evaluated using the Coverage Probability (CP) and the Average Length (AL). Let $\left(L^{\left(s\right)},U^{\left(s\right)}\right)$ denote the lower and upper bounds constructed in the *s*-th replication:

(60) $$\mathrm{CP}=\frac{1}{N_{rep}}\sum_{s=1}^{N_{rep}}I\left(L^{\left(s\right)}\leq Y_{obs}^{\left(s\right)}\leq U^{\left(s\right)}\right),\;\mathrm{AL}=\frac{1}{N_{rep}}\sum_{s=1}^{N_{rep}}\left(U^{\left(s\right)}-L^{\left(s\right)}\right),$$

where I(·) is the indicator function.

Table 1 compares the parameter estimation performance—measured by Root Mean Square Error (RMSE), Coverage Probability (CP), and Average Length (AL)—of the frequentist Maximum Likelihood Estimates (MLEs) and the Bayes estimates under both a non-informative prior (Prior 0) and the proposed empirical prior (Prior 1). As the distribution tail becomes heavier (i.e., as β decreases from 5.0 to 1.5), the estimation accuracy for the scale parameter α deteriorates, reflected by increased RMSEs across all methods. Nevertheless, the Bayes estimates under Prior 1 consistently yield the lowest RMSEs for both parameters across all censoring schemes, outperforming the frequentist MLEs and the Prior 0 Bayes estimates. For example, in the strict heavy-tailed regime (β=1.5, Scheme I), Prior 1 reduces the RMSE of α from 0.5183 (MLE) to 0.3724. This reduction demonstrates the regularization effect of the empirical prior in heavy-tailed settings. For interval estimation, Prior 1 produces substantially narrower credible intervals (e.g., the AL for β under Scheme II at β=5.0 decreases from 2.4402 under the frequentist approach to 1.7615) while maintaining empirical coverage probabilities near or slightly above the nominal 0.95 level.

Table 2 reports the RMSEs of the estimators across four proportional (n,m) settings with a fixed censoring proportion m/n=2/3. Overall, the estimation accuracy improves as the sample size increases. This pattern is also consistent with the findings of Barranco-Chamorro et al. [37]. For example, under the frequentist MLE, the RMSE of α decreases from 0.1434 to 0.1039 when β=5.0, from 0.3588 to 0.2599 when β=2.0, and from 0.4805 to 0.3473 when β=1.5 as (n,m) increases from (60,40) to (120,80). A similar monotone decline is observed for the estimation of β, with the corresponding RMSEs decreasing from 0.7180 to 0.4934, from 0.2872 to 0.1974, and from 0.2154 to 0.1480, respectively. Moreover, the Bayesian estimator under Prior 1 remains the most accurate method in nearly all settings. Its advantage is particularly evident for estimating the shape parameter β in the heavier-tailed regimes. For instance, when β=1.5, the RMSE of β under Prior 1 decreases from 0.1140 to 0.0951 across the four sample-size settings, whereas the corresponding values under the MLE are 0.2154, 0.1907, 0.1712, and 0.1480. Even at (n,m)=(120,80), Prior 1 still reduces the RMSE of β by about 35.7% relative to the MLE.

Table 3 examines the effect of reducing censoring intensity by increasing the observed-failure proportion m/n from 0.400 to 0.800 while keeping n=75 fixed. A clear feature of the results is that the gain is more pronounced for the estimation of the shape parameter β than for the scale parameter α. Under the frequentist MLE, the RMSE of α decreases moderately, from 0.1538 to 0.1207 when β=5.0, from 0.3806 to 0.3033 when β=2.0, and from 0.5069 to 0.4065 when β=1.5 as *m* increases from 30 to 60. In contrast, the corresponding RMSEs for β show a much larger reduction, falling from 0.9741 to 0.5865, from 0.3897 to 0.2346, and from 0.2922 to 0.1760, respectively. This indicates that the estimation of the tail-related shape parameter benefits particularly strongly from observing a larger proportion of failures, which is also in line with the general pattern reported by Barranco-Chamorro et al. [37]. Another notable point is that the Bayesian estimator under Prior 1 remains uniformly the most stable competitor across all m/n settings. For example, when β=1.5, its RMSE for β changes only from 0.1113 to 0.0982 as m/n increases, whereas the MLE decreases from 0.2922 to 0.1760 over the same range. Thus, increasing m/n improves all methods, but the empirical prior continues to provide substantial additional stabilization, especially in the heavier-tailed regimes.

Table 4 reports the biases and MSPEs of the point predictors under various progressive censoring schemes, prediction targets, and tail regimes (β). The proposed BP under Prior 1 yields the lowest bias magnitude and MSPE across most scenarios. In the finite-variance regime (Panel A, β=5.0), the frequentist BUP and the CMP perform comparably. However, in the strictly heavy-tailed regime (Panel C, β=1.5), the MSPEs for both the mean-based BUP and the median-based CMP increase substantially, though for distinct statistical reasons. This divergence stems from the extreme right-skewness of the distribution. For the BUP, the heavy right tail makes the mean highly sensitive to extreme observations, leading to a severe inflation in prediction variance. For the CMP, the right-skewness forces the conditional median to fall well below the conditional mean. While the median-based approach avoids variance inflation, it introduces a systematic downward bias (e.g., a bias of −5.5516 for the CMP compared to 0.4197 for the BUP in predicting the Mid-Min target under Scheme III). Under the bias-variance decomposition of MSPE, this persistent underestimation dominates the squared error term. Consequently, the overall prediction error of the CMP actually exceeds that of the BUP (614.8127 vs. 600.9724). In short, the high MSPE of the BUP is primarily driven by uncontrolled variance, whereas the CMP suffers from systematic underestimation. The performance improvement of the BP under Prior 1 over the BUP stems from two factors: the shift to a Bayesian framework and the injection of prior information. The Bayesian framework alone provides only marginal stability, as evidenced by the comparable performance of the BUP and the BP under the non-informative Prior 0. Therefore, the reduction in MSPE—such as the decrease from 2.9412 (BUP) to 2.3734 (Prior 1)—is primarily driven by the regularization effect of the empirical Bayes strategy rather than posterior integration alone. As expected, predicting further into the future (e.g., from Early-Min to Mid-Min) consistently increases prediction errors across all methods due to the greater uncertainty inherent in longer extrapolation horizons.

Table 5 summarizes the ALs and CPs for the five interval prediction methods. The frequentist PP method exhibits under-coverage for median targets, with CPs dropping to approximately 0.90. Conversely, all Bayesian methods maintain CPs near the nominal 0.95 level. The HPD methods consistently produce the shortest PIs. This advantage stems from how the ETI and HPD approaches handle the severe right-skewness of the Log-Logistic predictive distribution. Standard ETI methods trim an equal probability mass (γ/2) from both tails. Because the distribution features a steep left side and a heavy, flat right tail, symmetrically discarding γ/2 forces the upper bound to extend further into extreme values, yielding wider intervals. In contrast, HPD intervals minimize the total width by enforcing identical boundary densities (i.e., p(L)=p(U) for unimodal distributions). This constraint shifts the interval toward the mode, capturing the high-density region while omitting the low-density right tail. Consequently, the Bayes HPD method under Prior 1 reduces the AL (e.g., from 45.7393 under ETI to 27.6640 under HPD for the Mid-Min target in Panel C, Scheme III) without compromising the nominal coverage probability.

In summary, the selection of appropriate predictors depends strongly on the tail characteristics of the underlying data. For point prediction, the proposed BP under Prior 1 demonstrates the most stable and accurate overall performance across the evaluated scenarios. This advantage appears to arise primarily from the data-driven regularization induced by the empirical prior, rather than from Bayesian integration alone, as it helps stabilize prediction under heavy-tailed regimes and improves the overall bias–variance trade-off. When a frequentist procedure is preferred or the relevant moments are undefined, the CMP remains a moment-free alternative. However, under extremely heavy-tailed and strongly right-skewed predictive distributions, its practical performance may also deteriorate substantially due to systematic downward bias. For interval prediction, the Bayes HPD method under Prior 1 is the most effective strategy. By adapting to the right-skewness of the predictive distribution, it achieves shorter intervals while maintaining coverage probabilities close to the nominal level.

## 7. Real-Data-Based Validation

In this section, we conduct a real-data-based analysis using two complete survival datasets: the remission times of bladder cancer patients [38] and the survival times of guinea pigs [39]. For each dataset, we first examine the suitability of the Log-Logistic model and then evaluate the proposed prediction procedures under pre-specified progressive Type-II censoring schemes.

### 7.1. Example 1: Bladder Cancer Remission Data

The first dataset consists of the remission times (in months) of n=128 bladder cancer patients. Originally reported by Lee and Wang [38], this dataset is widely used in survival analysis to model right-skewed, heavy-tailed clinical data. The complete uncensored observations are provided in Table 6.

To justify the use of the Log-Logistic Distribution (LLD), we first assess its goodness-of-fit on the complete dataset. Fitting the LLD to these observations yields Maximum Likelihood Estimates (MLEs) of $\hat{\alpha }=6.0978$ and $\hat{\beta }=1.7160$. To rigorously verify its suitability, we compare the LLD against three standard alternative lifetime models: th-e Weibull, Lognormal, and Exponential distributions. Table 7 summarizes the log-likelihoods, information criteria (AIC, BIC), and several goodness-of-fit (GOF) statistics, including the Kolmogorov–Smirnov (K-S), Cramér–von Mises (CvM), Anderson–Darling (AD), Kuiper, and Watson tests.

**Figure 1:**
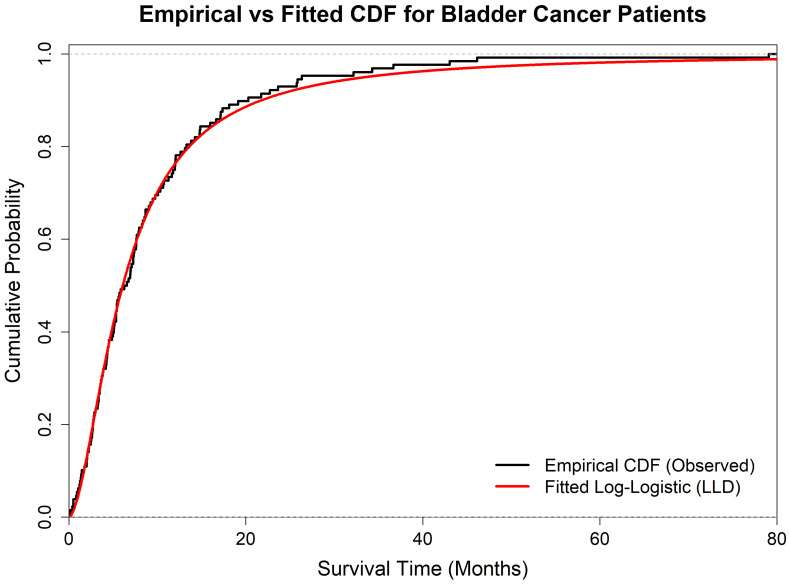
Empirical CDF overlaid with the fitted Log-Logistic CDF for the bladder cancer dataset.

As shown in Table 7, the LLD yields the highest log-likelihood and the lowest values across all information criteria and GOF statistics (e.g., K-S distance = 0.0399). These results indicate that the LLD provides the best fit for this dataset among the considered alternatives. Furthermore, the empirical cumulative distribution function (ECDF) overlaid with the fitted LLD CDF in Figure 1 illustrates a strong visual agreement with the data.

To generate random variates from the intractable full conditional distributions, we employ a Metropolis–Hastings (M-H) algorithm with a normal random-walk proposal, as detailed in Section 3. The initial values for the Markov chains are set to their respective Maximum Likelihood Estimates (MLEs), and the proposal variances are tuned using the inverse of the observed Fisher information. The algorithm is run for *N* = 10,000 iterations, with the first *B* = 2000 iterations discarded as burn-in. The acceptance rates for α and β are 42.63% and 48.22%, respectively, which fall well within the optimal range for random-walk Metropolis algorithms.

**Figure 2:**
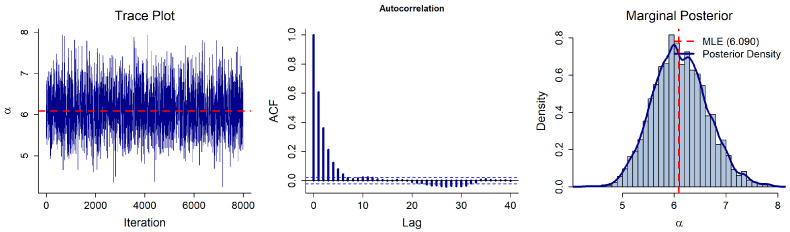
MCMC diagnostic plots for the scale parameter α.

**Figure 3:**
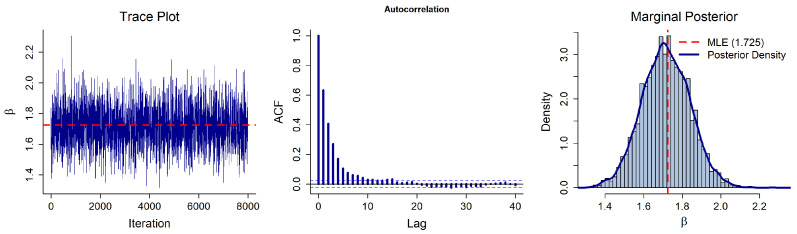
MCMC diagnostic plots for the shape parameter β.

Convergence of the MCMC algorithm is assessed using standard visual diagnostic tools: trace plots, autocorrelation function (ACF) plots, and marginal posterior histograms (Figure 2 and Figure 3). The trace plots exhibit random and patternless fluctuations around the central mass (near the MLEs, $\hat{\alpha }\approx 6.090$ and $\hat{\beta }\approx 1.725$), indicating adequate mixing. Furthermore, the ACF values decay to near zero within a lag of approximately 10, confirming low sample autocorrelation. Finally, the marginal posterior histograms are unimodal and reasonably symmetric. Together with the acceptance rates, these diagnostics suggest that the generated Markov chains have successfully converged to the stationary posterior distribution, providing a reliable basis for the subsequent Bayesian Predictions.

To evaluate prediction performance under controlled progressive Type-II censoring, we impose three pre-specified censoring schemes on the complete dataset and retain an effective observed sample size of m=64. Since the realized failure times of all units are available from the original complete sample, this construction also allows direct empirical assessment of point and interval prediction performance.

- Scheme I (Early Censoring): R=(20,15,15,10,4,0,…,0).
- Scheme II (Late Censoring): R=(0,…,0,4,10,15,15,20).
- Scheme III (Uniform Censoring): R=(1,1,…,1).

Table 8 and Table 9 present the point predictors (BUP, CMP, BP) and the 95% prediction intervals (PP, ETI, HPD), respectively, for various future targets. As an illustrative example under Scheme I, 20 surviving units are withdrawn at the first observed failure. To predict the 13th failure among these withdrawn units (i.e., $Y_{13:20:128}$), the point estimates are ${\hat{Y}}_{BUP}=8.528$, ${\hat{Y}}_{CMP}=8.175$, and ${\hat{Y}}_{BP}=8.643$. The corresponding 95% PIs are (4.89,14.21) for the frequentist PP, (4.71,15.14) for the Bayes ETI, and (4.11,13.76) for the Bayes HPD. All point predictors fall strictly within their respective intervals. Consistent with our simulation findings, the Bayes HPD method yields the shortest interval among the three interval procedures in this example. This suggests that HPD intervals may provide a more concise uncertainty summary for right-skewed survival outcomes under the censoring designs considered here.

### 7.2. Example 2: Guinea Pigs Survival Data

As a second real-data example, we consider the survival times (in days) of n=72 guinea pigs. This classical dataset, originally reported by Bjerkedal [39], is widely used in the biological assay and reliability literature. Similar to the bladder cancer data, these observations exhibit strong right-skewness, motivating the use of heavy-tailed models. The complete ordered data are provided in Table 10.

The suitability of the Log-Logistic Distribution (LLD) for this dataset was previously established by Maiti and Kayal [40]. Their analysis confirmed that the LLD provides a superior fit compared to classical models such as the Weibull and Exponential distributions, yielding a K-S distance of 0.0866. They reported the MLEs for the scale and shape parameters as $\hat{\alpha }=75.2745$ and $\hat{\beta }=2.5396$, respectively. Relying on this established validation, we proceed directly to the prediction task. Following a similar protocol to Example 1, we artificially censor the complete dataset to generate an effective sample of m=36 using three progressive Type-II censoring schemes:

- Scheme I (Early Censoring): R=(15,10,6,5,0,…,0).
- Scheme II (Late Censoring): R=(0,…,0,5,6,10,15).
- Scheme III (Uniform Censoring): R=(1,1,…,1).

Table 11 and Table 12 summarize the point and interval predictions across various future targets. To illustrate, consider the prediction of a deep-tail failure ($Y_{14:15:72}$) among the 15 units withdrawn at the first observed failure under Scheme I. The computed point estimates are ${\hat{Y}}_{BUP}=163.676$, ${\hat{Y}}_{CMP}=150.739$, and ${\hat{Y}}_{BP}=168.811$. All predictors are properly enveloped by their corresponding 95% prediction intervals: (92.36,311.82) for the frequentist PP, (89.24,343.39) for the Bayes ETI, and (78.15,294.74) for the Bayes HPD. Notably, the Bayes HPD interval effectively mitigates the severe right-tail overextension observed in the standard ETI method. This results in a substantially narrower prediction bound in the present example, again indicating that the HPD interval can be advantageous when summarizing predictive uncertainty for right-skewed survival data under the considered censoring schemes.

## 8. Conclusions

This paper investigated parameter estimation and the prediction of unobserved failure times for the Log-Logistic distribution under Progressive Type-II censoring. We developed point and interval prediction methods from both frequentist and Bayesian perspectives. For parameter estimation, Maximum Likelihood Estimators (MLEs) and Bayes estimates utilizing a Metropolis–Hastings within Gibbs algorithm were derived. We then constructed point predictors, including the Best Unbiased Predictor (BUP), Conditional Median Predictor (CMP), and Bayesian Predictor (BP), alongside interval predictors comprising frequentist Pivotal Prediction Intervals (PPI), Bayesian Equal-Tailed Intervals (ETI), and Highest Posterior Density (HPD) intervals.

The performance of these methods was evaluated through Monte Carlo simulations and validated using two real-world datasets (bladder cancer remission and guinea pig survival times). Based on the Bias and Mean Squared Prediction Error (MSPE), the proposed BP under the empirical prior (Prior 1) generally yields the most accurate point predictions. In contrast, under strict heavy-tailed regimes, the frequentist BUP and CMP may both deteriorate, although for different statistical reasons: the BUP is mainly affected by severe variance inflation, whereas the CMP is more prone to systematic downward bias under strong right-skewness.

For interval prediction, the Bayesian HPD method under Prior 1 yields the most precise intervals among the considered approaches. Because it does not force symmetric tail trimming, the HPD method substantially reduces the average interval length while maintaining the nominal coverage probability, demonstrating a clear practical advantage for inherently right-skewed survival data.

While this study focused on the Log-Logistic distribution under standard Progressive Type-II censoring, the proposed predictive frameworks can be extended to other flexible life-testing models, such as the Generalized Gamma or exponentiated non-monotonic hazard models. Future research could also generalize these prediction schemes to accommodate more complex censoring mechanisms, including adaptive progressive or unified hybrid Type-I/Type-II censoring schemes.

## Figures and Tables

**Table 1:** Comprehensive Parameter Estimation Performance (Point and Interval Metrics) across Different Tail Regimes.

Scheme	Method	Scale Parameter (α=3.0)					Shape Parameter (β∈{5.0,2.0,1.5})				
		Mean	SD	RMSE	CP	AL	Mean	SD	RMSE	CP	AL
Panel A: Light-Tailed Regime (True β=5.0)											
I (Early)	frequentist (MLE)	3.0084	0.1513	0.1515	0.941	0.5728	5.1005	0.6026	0.6107	0.940	2.2898
	Bayesian (Prior 0)	3.0146	0.1518	0.1525	0.945	0.5893	5.0523	0.5973	0.5993	0.933	2.2758
	Bayesian (Prior 1)	3.0136	0.1462	0.1468	0.943	0.5712	5.0188	0.3256	0.3259	0.992	1.6870
II (Late)	frequentist (MLE)	2.9991	0.1224	0.1224	0.951	0.4756	5.1601	0.6253	0.6452	0.961	2.4402
	Bayesian (Prior 0)	3.0080	0.1232	0.1234	0.958	0.4972	5.0873	0.6171	0.6229	0.954	2.4217
	Bayesian (Prior 1)	3.0048	0.1194	0.1194	0.960	0.4765	5.0912	0.3230	0.3355	0.988	1.7615
III (Uni)	frequentist (MLE)	2.9998	0.1319	0.1318	0.941	0.5094	5.1354	0.6177	0.6321	0.952	2.3340
	Bayesian (Prior 0)	3.0094	0.1329	0.1332	0.953	0.5287	5.0745	0.6108	0.6150	0.943	2.3188
	Bayesian (Prior 1)	3.0069	0.1275	0.1276	0.959	0.5105	5.0424	0.3271	0.3297	0.994	1.7072
Panel B: Infinite-Variance Boundary (True β=2.0)											
I (Early)	frequentist (MLE)	3.0353	0.3827	0.3841	0.943	1.4457	2.0402	0.2411	0.2443	0.940	0.9159
	Bayesian (Prior 0)	3.0647	0.3874	0.3926	0.945	1.5000	2.0210	0.2390	0.2398	0.931	0.9102
	Bayesian (Prior 1)	3.0524	0.3114	0.3156	0.966	1.3254	2.0089	0.1304	0.1306	0.990	0.6752
II (Late)	frequentist (MLE)	3.0071	0.3066	0.3065	0.948	1.1932	2.0640	0.2501	0.2581	0.961	0.9761
	Bayesian (Prior 0)	3.0398	0.3105	0.3129	0.964	1.2628	2.0348	0.2466	0.2489	0.956	0.9695
	Bayesian (Prior 1)	3.0226	0.2646	0.2654	0.972	1.1257	2.0378	0.1291	0.1344	0.989	0.7053
III (Uni)	frequentist (MLE)	3.0103	0.3299	0.3299	0.933	1.2798	2.0542	0.2471	0.2528	0.952	0.9336
	Bayesian (Prior 0)	3.0463	0.3353	0.3383	0.950	1.3438	2.0299	0.2446	0.2463	0.948	0.9272
	Bayesian (Prior 1)	3.0268	0.2781	0.2793	0.971	1.1934	2.0184	0.1306	0.1319	0.992	0.6831
Panel C: Extreme Heavy-Tailed Regime (True β=1.5)											
I (Early)	frequentist (MLE)	3.0579	0.5154	0.5183	0.942	1.9427	1.5301	0.1808	0.1832	0.940	0.6869
	Bayesian (Prior 0)	3.1086	0.5258	0.5366	0.948	2.0308	1.5160	0.1791	0.1797	0.931	0.6829
	Bayesian (Prior 1)	3.0754	0.3649	0.3724	0.979	1.6585	1.5071	0.0978	0.0980	0.989	0.5061
II (Late)	frequentist (MLE)	3.0164	0.4101	0.4102	0.942	1.5967	1.5480	0.1876	0.1936	0.961	0.7321
	Bayesian (Prior 0)	3.0677	0.4183	0.4235	0.965	1.7029	1.5263	0.1853	0.1870	0.958	0.7273
	Bayesian (Prior 1)	3.0328	0.3195	0.3210	0.976	1.4270	1.5290	0.0968	0.1010	0.989	0.5283
III (Uni)	frequentist (MLE)	3.0218	0.4411	0.4414	0.931	1.7143	1.5406	0.1853	0.1896	0.952	0.7002
	Bayesian (Prior 0)	3.0786	0.4524	0.4590	0.952	1.8184	1.5224	0.1833	0.1846	0.946	0.6955
	Bayesian (Prior 1)	3.0360	0.3307	0.3325	0.979	1.5048	1.5144	0.0978	0.0988	0.993	0.5119

**Table 2:** Sensitivity of parameter estimation accuracy to sample size under a fixed censoring proportion m/n=2/3 under the uniform progressive censoring scheme.

Method	(n,m)=(60,40)		(n,m)=(75,50)		(n,m)=(90,60)		(n,m)=(120,80)	
	α RMSE	β RMSE	α RMSE	β RMSE	α RMSE	β RMSE	α RMSE	β RMSE
Panel A: Light-Tailed Regime (True β=5.0)								
frequentist (MLE)	0.1434	0.7180	0.1316	0.6357	0.1181	0.5705	0.1039	0.4934
Bayesian (Prior 0)	0.1448	0.6919	0.1329	0.6188	0.1190	0.5573	0.1046	0.4843
Bayesian (Prior 1)	0.1378	0.3784	0.1273	0.3397	0.1152	0.3265	0.1019	0.3175
Panel B: Infinite-Variance Boundary (True β=2.0)								
frequentist (MLE)	0.3588	0.2872	0.3294	0.2543	0.2967	0.2282	0.2599	0.1974
Bayesian (Prior 0)	0.3691	0.2769	0.3376	0.2476	0.3028	0.2228	0.2640	0.1935
Bayesian (Prior 1)	0.2930	0.1517	0.2792	0.1357	0.2583	0.1305	0.2342	0.1269
Panel C: Extreme Heavy-Tailed Regime (True β=1.5)								
frequentist (MLE)	0.4805	0.2154	0.4408	0.1907	0.3977	0.1712	0.3473	0.1480
Bayesian (Prior 0)	0.5020	0.2078	0.4582	0.1855	0.4100	0.1670	0.3551	0.1452
Bayesian (Prior 1)	0.3425	0.1140	0.3339	0.1018	0.3135	0.0979	0.2891	0.0951

**Table 3:** Sensitivity of parameter estimation accuracy to the observed-failure proportion under the late progressive censoring scheme with fixed n=75. The table reports the RMSEs of the estimators for α and β across different values of m/n.

Method	m=30 (m/n=0.400)		m=40 (m/n=0.533)		m=50 (m/n=0.667)		m=60 (m/n=0.800)	
	α RMSE	β RMSE	α RMSE	β RMSE	α RMSE	β RMSE	α RMSE	β RMSE
Panel A: Light-Tailed Regime (True β=5.0)								
frequentist (MLE)	0.1538	0.9741	0.1298	0.7730	0.1224	0.6452	0.1207	0.5865
Bayesian (Prior 0)	0.1593	0.9114	0.1317	0.7379	0.1234	0.6229	0.1213	0.5721
Bayesian (Prior 1)	0.1341	0.3667	0.1234	0.3493	0.1194	0.3355	0.1183	0.3266
Panel B: Infinite-Variance Boundary (True β=2.0)								
frequentist (MLE)	0.3806	0.3897	0.3237	0.3092	0.3065	0.2581	0.3033	0.2346
Bayesian (Prior 0)	0.4147	0.3641	0.3369	0.2952	0.3129	0.2489	0.3077	0.2289
Bayesian (Prior 1)	0.2839	0.1478	0.2719	0.1396	0.2654	0.1344	0.2641	0.1311
Panel C: Extreme Heavy-Tailed Regime (True β=1.5)								
frequentist (MLE)	0.5069	0.2922	0.4323	0.2319	0.4102	0.1936	0.4065	0.1760
Bayesian (Prior 0)	0.5703	0.2733	0.4577	0.2216	0.4235	0.1870	0.4159	0.1715
Bayesian (Prior 1)	0.3324	0.1113	0.3271	0.1047	0.3210	0.1010	0.3196	0.0982

**Table 4:** Comprehensive Point Prediction Performance across Progressive Horizons and Tail Regimes.

Scheme	Target	Freq BUP (Mean)		Freq CMP (Median)		Bayes BP (Prior 0)		Bayes BP (Prior 1)	
		Bias	MSPE	Bias	MSPE	Bias	MSPE	Bias	MSPE
Panel A: Light-Tailed Regime (True β=5.0)									
I (Early)	Early-Min	0.0043	0.0725	−0.0146	0.0733	0.0018	0.0724	−0.0007	0.0694
	Early-Median	0.0033	0.0783	−0.0066	0.0782	0.0131	0.0788	0.0092	0.0767
II (Late)	Late-Min	−0.0017	0.0019	−0.0141	0.0020	0.0003	0.0019	−0.0001	0.0018
	Late-Median	−0.0128	0.0522	−0.0332	0.0521	0.0215	0.0539	0.0127	0.0429
III (Uni)	Early-Min	0.0022	1.4106	−0.2154	1.4492	0.0333	1.4132	0.0138	1.4063
	Mid-Min	−0.0518	1.5273	−0.3692	1.6354	−0.0026	1.5295	−0.0337	1.5011
Panel B: Infinite-Variance Boundary (True β=2.0)									
I (Early)	Early-Min	0.0097	0.0757	−0.0379	0.0768	0.0109	0.0757	0.0002	0.0714
	Early-Median	0.0228	0.5325	−0.0390	0.5268	0.0647	0.5419	0.0175	0.4811
II (Late)	Late-Min	−0.0038	0.0184	−0.0438	0.0200	0.0027	0.0185	0.0002	0.0180
	Late-Median	−0.0205	0.9157	−0.1349	0.8986	0.1389	0.9805	0.0667	0.7482
III (Uni)	Early-Min	0.2731	27.0435	−1.5208	28.5060	0.6164	27.6755	0.3081	26.5651
	Mid-Min	−0.1049	70.2079	−2.3702	73.9999	0.3993	71.2025	−0.0372	68.9627
Panel C: Extreme Heavy-Tailed Regime (True β=1.5)									
I (Early)	Early-Min	0.0104	0.0538	−0.0406	0.0545	0.0143	0.0538	−0.0006	0.0501
	Early-Median	0.0419	1.0117	−0.0696	0.9931	0.1073	1.0340	0.0071	0.8670
II (Late)	Late-Min	−0.0044	0.0428	−0.0662	0.0461	0.0034	0.0427	0.0004	0.0418
	Late-Median	−0.0030	2.9412	−0.2368	2.8445	0.2240	2.9547	0.1229	2.3734
III (Uni)	Early-Min	1.3347	131.7123	−3.2182	132.9556	2.2241	135.4824	1.3538	126.7600
	Mid-Min	0.4197	600.9724	−5.5516	614.8127	1.7133	604.7855	0.5089	589.8408

**Table 5:** Comprehensive Interval Prediction Performance across Progressive Horizons.

Scheme	Target	Frequentist PPI		Bayes ETI (Prior 0)		Bayes HPD (Prior 0)		Bayes ETI (Prior 1)		Bayes HPD (Prior 1)	
		CP	AL	CP	AL	CP	AL	CP	AL	CP	AL
Panel A: Light-Tailed Regime (True β=5.0)											
I (Early)	Early-Min	0.9350	0.9181	0.9460	0.9635	0.9450	0.8980	0.9510	0.9519	0.9460	0.8876
	Early-Median	0.9010	0.9348	0.9560	1.1302	0.9490	1.1070	0.9620	1.1118	0.9530	1.0906
II (Late)	Late-Min	0.9520	0.1486	0.9570	0.1599	0.9470	0.1303	0.9530	0.1567	0.9500	0.1281
	Late-Median	0.9040	0.7810	0.9600	0.9685	0.9510	0.9187	0.9630	0.8914	0.9640	0.8546
III (Uni)	Early-Min	0.9400	4.6917	0.9500	4.9456	0.9470	4.3259	0.9460	4.7963	0.9440	4.2291
	Mid-Min	0.9320	4.1228	0.9390	4.4153	0.9370	3.4687	0.9380	4.2329	0.9300	3.3452
Panel B: Infinite-Variance Boundary (True β=2.0)											
I (Early)	Early-Min	0.9350	0.9464	0.9460	1.0084	0.9440	0.8868	0.9510	0.9735	0.9440	0.8573
	Early-Median	0.9010	2.4293	0.9540	2.9802	0.9510	2.8481	0.9670	2.8174	0.9610	2.7068
II (Late)	Late-Min	0.9520	0.4751	0.9560	0.5124	0.9500	0.4150	0.9530	0.4971	0.9520	0.4045
	Late-Median	0.9040	3.2959	0.9590	4.2003	0.9510	3.8780	0.9650	3.7649	0.9640	3.5313
III (Uni)	Early-Min	0.9400	18.6156	0.9500	20.5111	0.9540	14.0789	0.9460	18.9057	0.9530	13.2354
	Mid-Min	0.9320	21.3236	0.9390	23.9913	0.9380	15.7864	0.9380	21.7348	0.9300	14.5996
Panel C: Extreme Heavy-Tailed Regime (True β=1.5)											
I (Early)	Early-Min	0.9350	0.7999	0.9460	0.8632	0.9460	0.7354	0.9520	0.8119	0.9440	0.6934
	Early-Median	0.9010	3.3437	0.9520	4.1169	0.9500	3.8538	0.9620	3.7833	0.9580	3.5698
II (Late)	Late-Min	0.9520	0.7280	0.9560	0.7757	0.9480	0.6264	0.9530	0.7566	0.9520	0.6128
	Late-Median	0.9040	5.9193	0.9600	7.3636	0.9520	6.7457	0.9670	6.7252	0.9640	6.2295
III (Uni)	Early-Min	0.9400	35.8651	0.9500	39.5406	0.9530	23.9910	0.9460	35.8224	0.9550	22.1636
	Mid-Min	0.9320	45.5940	0.9380	51.0883	0.9350	30.3131	0.9380	45.7393	0.9320	27.6640

**Table 6:** Real dataset representing the remission times (in months) of n=128 bladder cancer patients.

Bladder Cancer Remission Times											
0.08	0.20	0.40	0.50	0.51	0.81	0.90	1.05	1.19	1.26	1.35	1.40
1.46	1.76	2.02	2.02	2.07	2.09	2.23	2.26	2.46	2.54	2.62	2.64
2.69	2.69	2.75	2.83	2.87	3.02	3.25	3.31	3.36	3.36	3.48	3.52
3.57	3.64	3.70	3.82	3.88	4.18	4.23	4.26	4.33	4.34	4.40	4.50
4.51	4.87	4.98	5.06	5.09	5.17	5.32	5.32	5.34	5.41	5.41	5.49
5.62	5.71	5.85	6.25	6.54	6.76	6.93	6.94	6.97	7.09	7.26	7.28
7.32	7.39	7.59	7.62	7.63	7.66	7.87	7.93	8.26	8.37	8.53	8.65
8.66	9.02	9.22	9.47	9.74	10.06	10.34	10.66	10.75	11.25	11.64	11.79
11.98	12.02	12.03	12.07	12.63	13.11	13.29	13.80	14.24	14.76	14.77	14.83
15.96	16.62	17.12	17.14	17.36	18.10	19.13	20.28	21.73	22.69	23.63	25.74
25.82	26.31	32.15	34.26	36.66	43.01	46.12	79.05				

**Table 7:** Log-likelihood, Information Criteria, and Goodness-of-Fit Tests for the Bladder Cancer Dataset (n=128).

Model	Information Criteria			Goodness-of-Fit Statistics				
	LogLik	AIC	BIC	KS	CvM	AD	Kuiper	Watson
Log-Logistic	−411.4575	826.9151	832.6191	0.0399	0.0202	0.2454	0.0672	0.0202
Weibull	−414.0869	832.1738	837.8778	0.0700	0.1537	0.9580	0.1388	0.1468
Lognormal	−415.0944	834.1887	839.8928	0.0617	0.1186	0.8031	0.0970	0.0948
Exponential	−414.3419	830.6838	833.5358	0.0846	0.1788	1.1736	0.1454	0.1782

**Table 8:** Point Predictors under Different Censoring Schemes (Bladder Cancer Dataset).

Scheme I (Early Censoring)				Scheme II (Late Censoring)			
Target	BUP	CMP	BP	Target	BUP	CMP	BP
$Y_{1:20:128}$	0.965	0.859	0.967	$Y_{1:10:128}$	6.422	6.165	6.442
$Y_{7:20:128}$	4.119	3.992	4.131	$Y_{4:10:128}$	9.778	9.279	9.906
$Y_{13:20:128}$	8.528	8.175	8.643	$Y_{6:10:128}$	13.738	12.786	14.010
$Y_{19:20:128}$	30.304	25.097	31.658	$Y_{9:10:128}$	34.093	27.573	35.921
$Y_{1:15:128}$	1.186	1.049	1.193	$Y_{1:15:128}$	6.383	6.215	6.398
$Y_{5:15:128}$	3.923	3.763	3.928	$Y_{5:15:128}$	9.161	8.866	9.243
$Y_{10:15:128}$	8.841	8.347	8.909	$Y_{10:15:128}$	16.322	15.408	16.776
$Y_{14:15:128}$	25.303	20.924	26.499	$Y_{14:15:128}$	45.492	36.819	48.780
$Y_{1:15:128}$	1.279	1.134	1.273	$Y_{1:15:128}$	6.791	6.620	6.805
$Y_{5:15:128}$	3.974	3.811	3.991	$Y_{5:15:128}$	9.624	9.320	9.692
$Y_{10:15:128}$	8.896	8.400	8.998	$Y_{10:15:128}$	16.974	16.030	17.482
$Y_{14:15:128}$	25.414	21.017	26.560	$Y_{14:15:128}$	47.048	38.092	50.138
$Y_{1:10:128}$	1.901	1.690	1.909	$Y_{1:20:128}$	6.948	6.820	6.958
$Y_{4:10:128}$	4.839	4.551	4.874	$Y_{7:20:128}$	10.218	9.970	10.345
$Y_{6:10:128}$	7.635	7.096	7.709	$Y_{13:20:128}$	16.927	16.236	17.326
$Y_{9:10:128}$	19.891	16.406	20.414	$Y_{19:20:128}$	58.121	47.069	62.621
**Scheme III (Uniform Censoring)**							
$Y_{1:1:128}$	13.453	6.349	14.420	$Y_{1:1:128}$	19.015	9.766	20.647
$Y_{1:1:128}$	14.838	7.197	16.184	$Y_{1:1:128}$	20.800	10.854	22.197
$Y_{1:1:128}$	16.161	8.013	17.279	$Y_{1:1:128}$	25.656	13.788	27.678
$Y_{1:1:128}$	17.455	8.810	19.016	$Y_{1:1:128}$	38.443	21.392	41.043

Note: repeated targets under Scheme III correspond to different censoring stages selected from the uniform censoring scheme.

**Table 9:** Ninety-Five Percent Prediction Intervals under Different Censoring Schemes (Bladder Cancer Dataset).

Scheme I (Early Censoring)				Scheme II (Late Censoring)			
Target	PP	Bayes ETI	Bayes HPD	Target	PP	Bayes ETI	Bayes HPD
$Y_{1:20:128}$	(0.15, 2.38)	(0.14, 2.47)	(0.08, 2.27)	$Y_{1:10:128}$	(5.64, 8.66)	(5.64, 8.78)	(5.62, 8.03)
$Y_{7:20:128}$	(2.24, 6.73)	(2.12, 7.00)	(1.68, 6.39)	$Y_{4:10:128}$	(6.65, 15.80)	(6.64, 16.54)	(6.15, 14.63)
$Y_{13:20:128}$	(4.89, 14.21)	(4.71, 15.14)	(4.11, 13.76)	$Y_{6:10:128}$	(8.10, 24.96)	(8.03, 26.82)	(7.11, 22.14)
$Y_{19:20:128}$	(11.66, 80.12)	(11.27, 89.27)	(7.20, 73.35)	$Y_{9:10:128}$	(12.95, 94.26)	(12.77, 105.79)	(9.46, 82.19)
$Y_{1:15:128}$	(0.26, 2.89)	(0.26, 2.98)	(0.21, 2.77)	$Y_{1:15:128}$	(5.86, 7.85)	(5.86, 7.94)	(5.85, 7.60)
$Y_{5:15:128}$	(1.89, 6.88)	(1.79, 7.12)	(1.56, 6.51)	$Y_{5:15:128}$	(6.85, 13.17)	(6.83, 13.72)	(6.81, 12.89)
$Y_{10:15:128}$	(4.62, 15.93)	(4.44, 16.79)	(3.48, 15.20)	$Y_{10:15:128}$	(9.97, 28.01)	(9.84, 30.69)	(9.38, 28.42)
$Y_{14:15:128}$	(9.51, 67.30)	(9.28, 74.93)	(6.84, 63.54)	$Y_{14:15:128}$	(17.25, 125.64)	(16.77, 146.56)	(11.36, 118.96)
$Y_{1:15:128}$	(0.44, 2.94)	(0.44, 2.99)	(0.40, 2.57)	$Y_{1:15:128}$	(6.26, 8.28)	(6.26, 8.37)	(6.25, 7.86)
$Y_{5:15:128}$	(1.95, 6.93)	(1.88, 7.18)	(1.72, 6.39)	$Y_{5:15:128}$	(7.27, 13.73)	(7.25, 14.20)	(6.98, 12.96)
$Y_{10:15:128}$	(4.67, 16.01)	(4.53, 16.85)	(4.27, 15.39)	$Y_{10:15:128}$	(10.45, 29.01)	(10.30, 31.93)	(9.61, 28.43)
$Y_{14:15:128}$	(9.57, 67.58)	(9.30, 75.18)	(7.02, 59.97)	$Y_{14:15:128}$	(17.92, 129.77)	(17.44, 149.97)	(13.75, 116.16)
$Y_{1:10:128}$	(0.94, 4.06)	(0.93, 4.13)	(0.90, 3.72)	$Y_{1:20:128}$	(6.55, 8.07)	(6.55, 8.13)	(6.54, 7.87)
$Y_{4:10:128}$	(2.24, 9.11)	(2.19, 9.44)	(1.97, 8.44)	$Y_{7:20:128}$	(7.92, 13.94)	(7.89, 14.63)	(7.73, 13.48)
$Y_{6:10:128}$	(3.59, 14.83)	(3.50, 15.52)	(3.25, 13.63)	$Y_{13:20:128}$	(11.19, 26.67)	(11.00, 29.15)	(9.94, 26.50)
$Y_{9:10:128}$	(7.21, 53.44)	(7.05, 57.31)	(4.10, 44.04)	$Y_{19:20:128}$	(22.14, 160.25)	(21.41, 188.20)	(16.35, 140.62)
**Scheme III (Uniform Censoring)**							
$Y_{1:1:128}$	(0.89, 62.17)	(0.87, 66.03)	(0.53, 56.75)	$Y_{1:1:128}$	(4.37, 80.67)	(4.37, 87.37)	(4.18, 54.55)
$Y_{1:1:128}$	(1.99, 66.58)	(1.99, 71.94)	(1.77, 42.23)	$Y_{1:1:128}$	(5.25, 86.89)	(5.25, 92.60)	(5.07, 53.20)
$Y_{1:1:128}$	(2.82, 70.95)	(2.82, 75.48)	(2.62, 44.73)	$Y_{1:1:128}$	(7.49, 104.12)	(7.49, 112.63)	(7.28, 52.24)
$Y_{1:1:128}$	(3.55, 75.32)	(3.56, 82.07)	(3.37, 55.81)	$Y_{1:1:128}$	(12.90, 150.64)	(12.90, 161.29)	(12.64, 102.10)

**Table 10:** Real dataset representing the survival times (in days) of n=72 guinea pigs.

Guinea Pigs Survival Times											
12	15	22	24	24	32	32	33	34	38	38	43
44	48	52	53	54	54	55	56	57	58	58	59
60	60	60	60	61	62	63	65	65	67	68	70
70	72	73	75	76	76	81	83	84	85	87	91
95	96	98	99	109	110	121	127	129	131	143	146
146	175	175	211	233	258	258	263	297	341	341	376

**Table 11:** Point Predictors under Different Censoring Schemes (Guinea Pigs Dataset).

Scheme I (Early Censoring)				Scheme II (Late Censoring)			
Target	BUP	CMP	BP	Target	BUP	CMP	BP
$Y_{1:15:72}$	25.319	24.245	25.382	$Y_{1:5:72}$	74.064	71.469	74.361
$Y_{7:15:72}$	64.811	63.771	64.984	$Y_{3:5:72}$	99.852	95.447	101.378
$Y_{14:15:72}$	163.676	150.739	168.811	$Y_{4:5:72}$	123.979	115.809	128.110
$Y_{1:10:72}$	30.058	28.623	30.212	$Y_{1:6:72}$	75.448	73.275	75.773
$Y_{5:10:72}$	67.390	65.726	67.851	$Y_{3:6:72}$	95.098	91.916	96.746
$Y_{9:10:72}$	139.026	127.992	142.503	$Y_{5:6:72}$	135.453	126.621	139.271
$Y_{1:6:72}$	39.815	37.471	39.862	$Y_{1:10:72}$	74.463	73.141	74.643
$Y_{3:6:72}$	67.631	64.896	68.110	$Y_{5:10:72}$	98.135	96.053	99.607
$Y_{5:6:72}$	113.162	104.072	115.056	$Y_{9:10:72}$	163.844	153.310	169.140
$Y_{1:5:72}$	42.082	39.448	42.271	$Y_{1:15:72}$	72.996	72.102	73.166
$Y_{3:5:72}$	76.476	72.401	77.364	$Y_{7:15:72}$	96.108	94.759	97.068
$Y_{4:5:72}$	103.882	95.469	105.439	$Y_{14:15:72}$	187.577	175.567	195.693
**Scheme III (Uniform Censoring)**							
$Y_{1:1:72}$	81.399	70.232	82.668	$Y_{1:1:72}$	113.045	96.661	115.402
$Y_{1:1:72}$	93.508	79.679	95.343	$Y_{1:1:72}$	142.035	122.153	144.575

**Table 12:** Ninety-Five Percent Prediction Intervals under Different Censoring Schemes (Guinea Pigs Dataset).

Scheme	Target	PPI	Bayes ETI	Bayes HPD
I (Early)	$Y_{1:15:72}$	(12.86, 44.27)	(12.78, 45.63)	(12.03, 42.69)
	$Y_{7:15:72}$	(43.77, 91.84)	(41.81, 96.29)	(39.92, 90.56)
	$Y_{14:15:72}$	(92.36, 311.82)	(89.24, 343.39)	(78.15, 294.74)
	$Y_{1:10:72}$	(15.89, 52.79)	(15.84, 54.21)	(15.13, 50.85)
	$Y_{5:10:72}$	(41.88, 102.54)	(40.49, 107.39)	(35.29, 99.44)
	$Y_{9:10:72}$	(76.33, 267.27)	(74.27, 289.28)	(65.01, 255.75)
	$Y_{1:6:72}$	(24.70, 68.42)	(24.67, 69.67)	(24.09, 62.79)
	$Y_{3:6:72}$	(38.36, 112.92)	(37.69, 117.27)	(37.33, 113.27)
	$Y_{5:6:72}$	(59.35, 221.01)	(58.03, 235.06)	(43.78, 195.24)
	$Y_{1:5:72}$	(24.84, 74.64)	(24.81, 76.44)	(24.09, 72.32)
	$Y_{3:5:72}$	(41.06, 135.90)	(40.39, 142.47)	(36.44, 135.35)
	$Y_{4:5:72}$	(52.95, 204.85)	(51.87, 216.73)	(38.53, 179.55)
II (Late)	$Y_{1:5:72}$	(65.24, 97.36)	(65.24, 99.26)	(65.02, 93.04)
	$Y_{3:5:72}$	(72.37, 153.04)	(72.30, 161.48)	(67.87, 144.63)
	$Y_{4:5:72}$	(80.07, 216.21)	(80.00, 237.44)	(71.80, 210.02)
	$Y_{1:6:72}$	(68.20, 94.80)	(68.20, 96.70)	(68.00, 93.24)
	$Y_{3:6:72}$	(73.73, 134.87)	(73.74, 142.87)	(71.02, 133.60)
	$Y_{5:6:72}$	(87.54, 235.60)	(87.09, 256.98)	(78.33, 242.01)
	$Y_{1:10:72}$	(70.12, 86.16)	(70.12, 87.24)	(70.00, 83.72)
	$Y_{5:10:72}$	(79.21, 129.05)	(79.04, 136.17)	(75.16, 128.45)
	$Y_{9:10:72}$	(105.05, 284.95)	(103.72, 315.67)	(96.83, 306.62)
	$Y_{1:15:72}$	(70.08, 80.89)	(70.08, 81.82)	(70.00, 78.92)
	$Y_{7:15:72}$	(80.59, 119.35)	(80.18, 125.16)	(76.78, 119.03)
	$Y_{14:15:72}$	(119.84, 326.37)	(117.52, 370.94)	(108.36, 326.85)
III (Uni)	$Y_{1:1:72}$	(25.25, 205.66)	(24.80, 215.44)	(17.27, 164.28)
	$Y_{1:1:72}$	(49.60, 220.95)	(49.61, 232.60)	(48.03, 188.89)
	$Y_{1:1:72}$	(71.04, 252.60)	(71.05, 267.09)	(70.10, 221.85)
	$Y_{1:1:72}$	(95.96, 306.20)	(95.97, 321.95)	(95.18, 270.85)

## Data Availability

The datasets are provided in refs. [38,39].

## References

[1] Lawless J.F. (2003). Statistical Models and Methods for Lifetime Data.

[2] Kundu D., Raqab M.Z. (2012). Bayesian inference and prediction of order statistics for a Type-II censored Weibull distribution. J. Stat. Plan. Inference.

[3] Almalki S.J., Nadarajah S. (2014). Modifications of the Weibull distribution: A review. Reliab. Eng. Syst. Saf..

[4] Fisk P.R. (1961). The graduation of income distributions. Econometrica.

[5] Nelson W. (1982). Applied Life Data Analysis.

[6] Clavijo-Blanco J.A., González-Cagigal M.A., Rosendo-Macías J.A. (2024). Statistical characterization of reliability indices in medium voltage networks using a Monte Carlo-based method. Electr. Power Syst. Res..

[7] Khan M.Y., Iqbal T., Iqbal T., Shah M.A. (2020). Probabilistic modeling of earthquake interevent times in different regions of Pakistan. Pure Appl. Geophys..

[8] Ashkar F., Mahdi S. (2006). Fitting the Log-Logistic distribution by generalized moments. J. Hydrol..

[9] Bennett S. (1983). Log-Logistic regression models for survival data. J. R. Stat. Soc. Ser. C (Appl. Stat.).

[10] Gupta R.C., Akman O., Lvin S. (1999). A study of Log-Logistic model in survival analysis. Biom. J..

[11] O’Quigley J., Struthers L. (1982). Survival models based upon the logistic and Log-Logistic distributions. Comput. Programs Biomed..

[12] Balakrishnan N., Malik H.J. (1987). Best linear unbiased estimation of the location and scale parameters of the Log-Logistic distribution. Commun. Stat.-Theory Methods.

[13] Al-Shomrani A.A., Shawky A.I., Arif O.H., Aslam M. (2016). Log-Logistic distribution for survival data analysis using MCMC. SpringerPlus.

[14] Abbas K., Tang Y. (2016). Objective Bayesian analysis for Log-Logistic distribution. Commun. Stat.-Simul. Comput..

[15] Howlader H.A., Weiss G. (1992). Log-Logistic survival estimation based on failure-censored data. J. Appl. Stat..

[16] Kantam R.R.L., Rao G.S., Sriram B. (2006). An economic reliability test plan: Log-Logistic distribution. J. Appl. Stat..

[17] Hyun S., Lee J., Yearout R.D. (2016). Parameter estimation of Type-I and Type-II hybrid censored data from the Log-Logistic distribution. Ind. Syst. Eng. Rev..

[18] Balakrishnan N., Aggarwala R. (2000). Progressive Censoring: Theory, Methods, and Applications.

[19] Ng H.K.T., Chan P.S., Balakrishnan N. (2002). Estimation of parameters from progressively censored data using EM algorithm. Comput. Stat. Data Anal..

[20] Wu S.J. (2002). Estimations of the parameters of the Weibull distribution with progressively censored data. J. Jpn. Stat. Soc..

[21] Wu S.F., Wu C.C., Chen Y.L., Yu Y.R., Lin Y.P. (2010). Interval estimation of a two-parameter Burr-XII distribution under progressive censoring. Statistics.

[22] Mousa M.A.M.A., Jaheen Z.F. (2002). Bayesian Prediction for progressively censored data from the Burr model. Stat. Pap..

[23] Raqab M.Z., Asgharzadeh A., Valiollahi R. (2010). Prediction for Pareto distribution based on progressively Type-II censored samples. Comput. Stat. Data Anal..

[24] Wu S.J. (2008). Estimation of the two-parameter bathtub-shaped lifetime distribution with progressive censoring. J. Appl. Stat..

[25] Pradhan B., Kundu D. (2009). On progressively censored generalized exponential distribution. TEST.

[26] Yadav A.S., Singh S.K., Singh U. (2018). Estimation of stress–strength reliability for inverse Weibull distribution under progressive type-II censoring scheme. J. Ind. Prod. Eng..

[27] Mäkeläinen T., Schmidt K., Styan G.P.H. (1981). On the existence and uniqueness of the maximum likelihood estimate of a vector-valued parameter in fixed-size samples. Ann. Stat..

[28] Lawless J.F. (1982). Statistical Models and Methods for Lifetime Data.

[29] Lin C.T., Balakrishnan N. (2011). Asymptotic properties of maximum likelihood estimators based on progressive Type-II censoring. Metrika.

[30] David H.A., Nagaraja H.N. (2003). Order Statistics.

[31] Chen M.H., Shao Q.M. (1999). Monte Carlo estimation of Bayesian credible and HPD intervals. J. Comput. Graph. Stat..

[32] Abou Ghaida W.R., Baklizi A. (2022). Prediction of future failures in the Log-Logistic distribution based on hybrid censored data. Int. J. Syst. Assur. Eng. Manag..

[33] Clark D.E., El-Taha M. (2015). Some Useful Properties of Log-Logistic Random Variables for Health Care Simulations. Int. J. Stat. Med. Res..

[34] Kleiber C., Kotz S. (2003). Statistical Size Distributions in Economics and Actuarial Sciences.

[35] Johnson N.L., Kotz S., Balakrishnan N. (1995). Continuous Univariate Distributions, Volume 2.

[36] Resnick S.I. (2007). Heavy-Tail Phenomena: Probabilistic and Statistical Modeling.

[37] Barranco-Chamorro I., Luque-Calvo P.L., Jiménez-Gamero M.D., Alba-Fernández M.V. (2017). A study of risks of Bayes estimators in the generalized half-logistic distribution for progressively type-II censored samples. Math. Comput. Simul..

[38] Lee E.T., Wang J.W. (2003). Statistical Methods for Survival Data Analysis.

[39] Bjerkedal T. (1960). Acquisition of resistance in guinea pigs infected with different doses of virulent tubercle bacilli. Am. J. Epidemiol..

[40] Maiti K., Kayal S. (2023). Estimating reliability characteristics of the Log-Logistic distribution under progressive censoring with two applications. Ann. Data Sci..

